# Catalytic insertion of E–H bonds (E = C, N, P, S) into heterocumulenes by amido–actinide complexes†
†Electronic supplementary information (ESI) available: Detailed experimental conditions, product characterisation, kinetic, and thermodynamic data are provided. See DOI: 10.1039/c5sc02746b


**DOI:** 10.1039/c5sc02746b

**Published:** 2015-10-29

**Authors:** Rami J. Batrice, Moris S. Eisen

**Affiliations:** a Schulich Faculty of Chemistry , Technion – Israel Institute of Technology , Technion City , 32000 , Haifa , Israel . Email: chmoris@tx.technion.ac.il

## Abstract

We report herein the actinide-mediated insertion of E–H bonds (E = C, N, P, S) into various heterocumulenes including carbodiimides, isocyanates, and isothiocyanates. The precatalysts are prepared by a simple, one-pot procedure using readily available starting materials, and challenging insertions are achieved with excellent selectivity in short reaction times. Spectroscopic data are utilised to propose the active catalytic mechanism and derive thermodynamic activation parameters, which are described in this study. The only example of an actinide-mediated C–H bond insertion into a carbon–heteroatom bond is presented within this study.

## Abstract 2

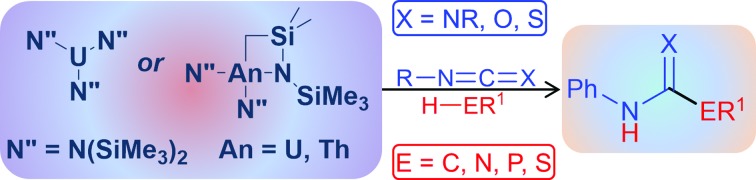
We report herein the actinide-mediated insertion of E–H bonds (E = C, N, P, S) into various heterocumulenes including carbodiimides, isocyanates, and isothiocyanates.

## Introduction

The structural and catalytic behaviour of the actinides has attracted considerable attention in recent years owing to their unique coordination geometries,^1^–^10^ as well as their impressive reactivity profiles in numerous stoichiometric and catalytic processes. Hydroaminations,^11^–^18^ hydroalkoxylations,^19^ hydrosilylations,^20^–^22^ polymerisations,^23^–^29^ alkyne oligomerisations,^30^–^32^ and numerous small molecule activations^28^,^33^–^40^ represent a few of the advancements made in this field. However, the insertion of heterocumulenes into E–H bonds (E = N, P, S) has only recently appeared in the scope of accessible reactions in f-block chemistry.^41^–^47^ The guanidine, phosphaguanidine, and thiourea products obtained have found considerable utility as ligands in coordination compounds,^48^ medicinal applications,^49^ and synthons for challenging organic transformations.^50^

**Fig. 1:**
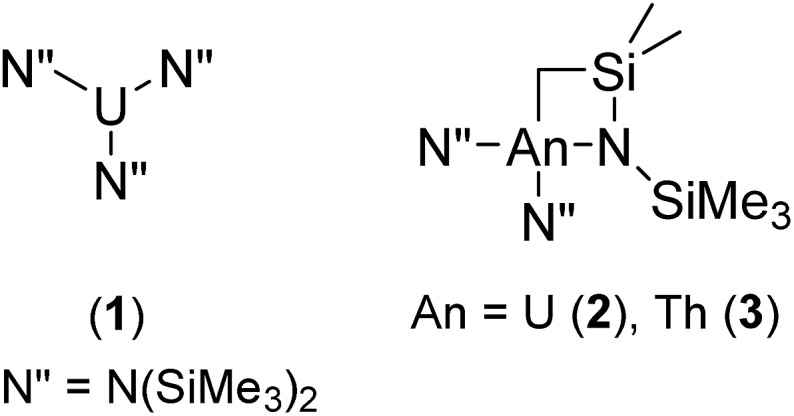
Precatalysts employed for the catalytic insertion of E–H bonds into heterocumulenes.

Carbodiimides in particular have been shown to insert stoichiometrically into the actinide–carbon bond of Cp*2AnMe_2_ (An = U, Th), giving rise to the corresponding pentamethylcyclopentadienyl–amidinate complexes of the early actinides,^51^ however only in this year was the process made catalytic, mediated by a mono(imidazolin-2-iminato) thorium(iv) complex and often accompanied by extended reaction times and moderate conversions.^52^ Moreover, the low-yielding multi-step ligand synthesis provides a further challenge accompanying the production and application of this complex. Recently, we have shown that a series of simple amido–actinide complexes of the formula U[N(SiMe_3_)_2_]_3_ (**1**) and [(Me_3_Si)_2_N]_2_An[κ^2^-(*N*,*C*)–CH_2_Si(CH_3_)_2_N(SiMe_3_)] (An = U (**2**), Th (**3**)) (Fig. 1) are capable of effecting the efficient oligomerisation or cyclotrimerisation of terminal alkynes,^53^ the sole example of a catalytic transformation employing this class of complexes prior to the work described in this study. These complexes are generated in a one-pot process using readily available starting materials and have already shown impressive stoichiometric reactivity,^54^,^55^ making them exceptionally attractive in their use as catalysts. In our attempt to uncover new reactivity with this class of complexes, we have applied them toward the catalytic insertion of E–H bonds into heterocumulenes (Y = C=X), wherein E = C, N, P, or S, and Y = RN, and X = RN, O, or S. Most interestingly was the catalytic insertion of C–H bonds into carbodiimides, the only example of actinide-mediated C–H insertion into a C–X unsaturated bond to date.

Previously, the effects of opening the coordination sphere of an actinide catalyst was achieved in cyclopentadienyl–actinide systems by the use of an *ansa*-bridged tetramethylcyclopentadienyl ligand, resulting in markedly increased activity and selectivity.^56^ In the catalytic study of the amido–actinide complexes (**1–3**), the coordinative unsaturation is considerably increased as compared to the analogous actinide(imidazolin-2-iminato) complex, similarly providing superior reactivity over the previously reported system. These findings open the scope of possible catalytic transformations toward new, challenging reactions.

## Results and discussion

**Fig. 2:**
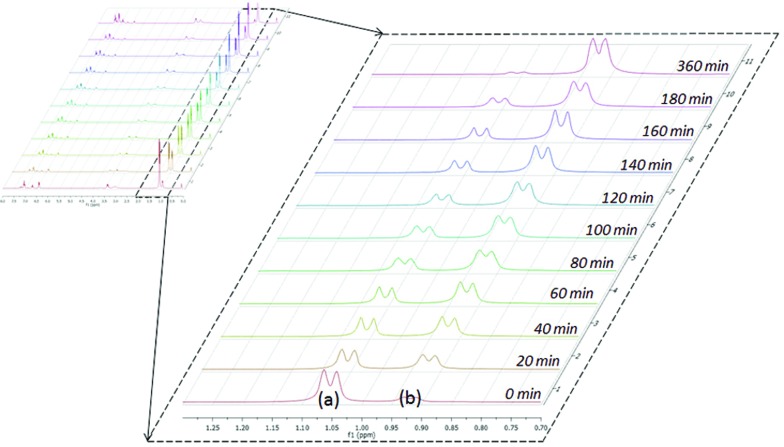
Reaction progress of aniline insertion into DIC by complex **1** at 50 °C; DIC consumption (a) and guanidine formation (b).

**Fig. 3:**
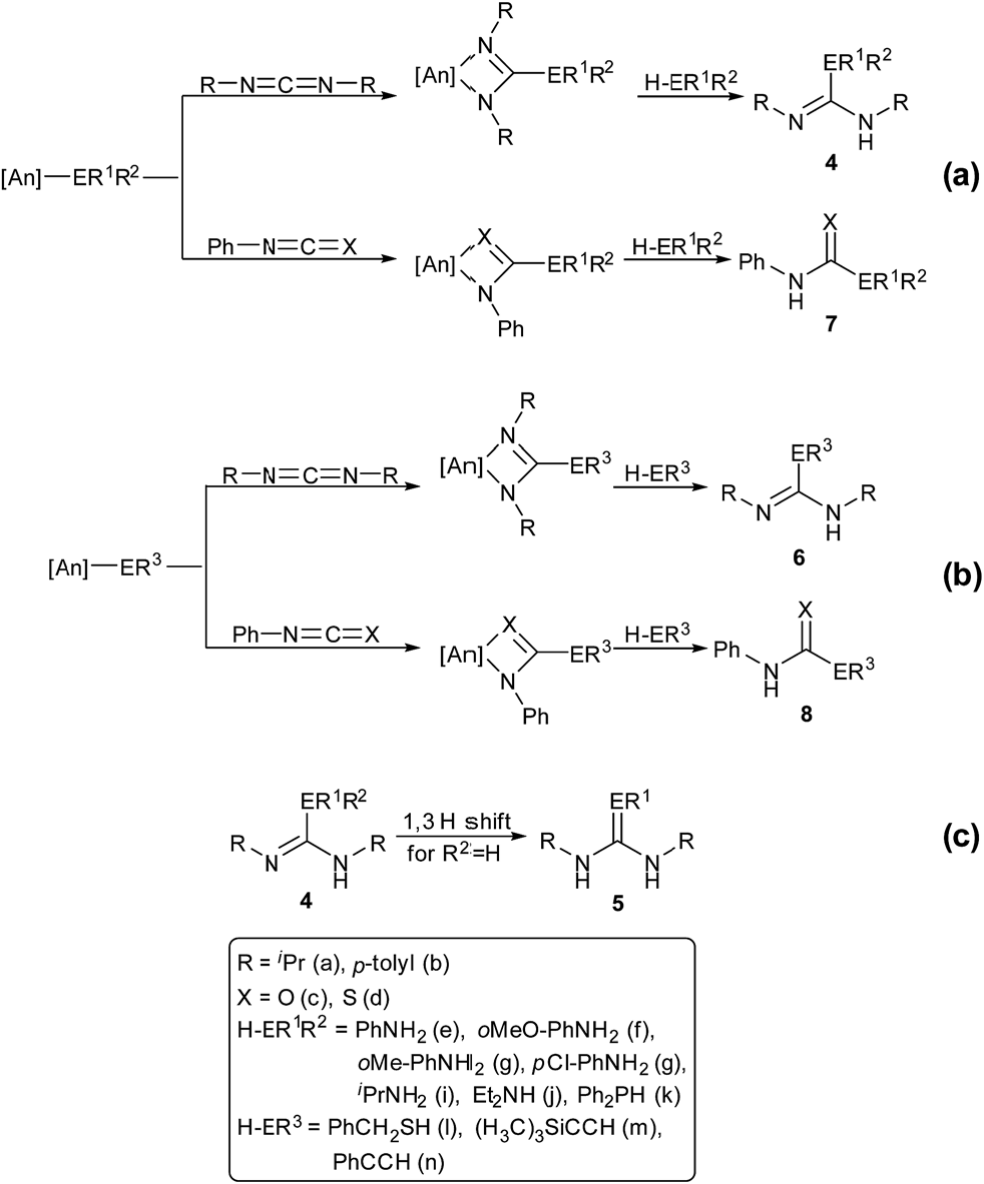
Catalytic insertion of E–H into heterocumulenes mediated by precatalysts **1–3**.

The catalytic reactions of *N*,*N*′-diisopropylcarbodiimide (DIC), di-*p*-tolylcarbodiimide (DTC), phenylisocyanate, and phenylisothiocyanate with a series REH moieties are presented below. To establish the potential reactivity of these complexes, the insertion of aniline into diisopropylcarbodiimide was monitored using ^1^H NMR spectroscopy (Fig. 2), showing the reaction progress as a function of DIC consumption and guanidine formation, (Fig. 2(a) and (b), respectively). Further investigation of the mode of activation of the actinide centre was performed by independent addition of aniline and DIC. In each of the uranium and thorium complexes, it is seen that complete displacement of the hexamethyldisilazane occurs after the addition of aniline, and that conservation of the precatalyst oxidation state is maintained throughout the reaction. Conversely, the reaction with DIC necessitated elevated temperatures to activate the metal centre. These findings inform us that activation of the precatalyst is achieved by the amine moiety to generate the catalytically active species. Attempts to isolate the intermediates generated and characterisation by crystallisation were unsuccessful, however previous studies have shown that activation of the thorium complex **3** with terminal alkyne similarly results in complete displacement of the amido moieties.^53^ It is worth note that the order of addition of the reagents to the precatalyst solution did not markedly affect the reaction rate or product distribution. The nature of the REH substrate additionally affects the outcome of the insertion product (Fig. 3); where E is a monoprotic nucleophile, product **4** is obtained as the sole insertion product. However, when E is a diprotic nucleophile (such as a primary amine), isomerisation of compound **4** is possible, yielding product **5** as the major product. The isomerisation with diprotic nucleophiles is dependent on the resulting guanidine generated, wherein the formation of the C=N bond is directed toward the more electron withdrawing arm of the structure. The insertion of a series of REH moieties into diisopropylcarbodiimide, mediated by the three actinide complexes presented (**1–3**), was investigated and the results summarised in Table 1. All experiments presented herein were performed in the presence of 100 equivalents of heterocumulene and REH moiety. It is seen that complexes **1–3** efficiently mediate the insertion of various anilines, effecting the transformation to the desired guanidines in excellent yield within 4–12 hours (Table 1, entries 1–12). Each of the products was detected as the analogous product **5** by tautomerisation forming the *ipso*-C=E bond (Fig. 3(c)). When aliphatic amines were employed, the conversion is markedly decreased, yielding at best 31% of 1,1-diethyl-2,3-diisopropylguanidine (Table 1, entry 16), indicating that the formation of the actinide–aliphatic amine bond is more energetically stable and less susceptible to insertion by the carbodiimide. Using diphenylphosphine as the REH species showed moderate conversions to the phosphaguanidine using catalysts **1** and **2** (Table 1, entries 17 and 18). Particularly impressive, however, was the quantitative conversion of phosphine to the desired product in 2 hours by the thorium metallacycle **3** (Table 1, entry 19). The substantially higher activity of the thorium precatalyst (**3**) is an outcome of the electron deficiency of the metal centre. As the electrophilicity of the metal centre increases, the protonolysis by the nucleophilic phosphine is accelerated. The 5f electrons of the uranium catalysts decreases electrophilicity at the metal, but additionally participate in π-back-bonding interactions to phosphine, inhibiting the catalytic turnover, whereas the thorium complex (**3**) (lacking 5f electrons) is unable to perform this stabilising interaction and allows for rapid protonolysis and insertion. The insertion of benzylthiol was attempted with each of the uranium precatalysts, however upon addition of the thiol, immediate formation of a green precipitate was observed, which reacted no further after heating. The thorium complex **3**, conversely, reacted within two hours to near-quantitative conversion toward product **6al** (Table 1, entry 20). It is interesting to note the lower activity of the thorium complex (**3**) as compared to the uranium complexes employed in the reaction with *ortho*-anisidine (Table 1, entries 4–6). It is proposed that the highly electrophilic thorium catalyst forms a dative coordination to the *o*-methoxy functionality of the aniline, thus inhibiting the catalytic turnover.

**Table 1:** Catalytic reactions of diisopropylcarbodiimide with E–H species mediated by complexes **1–3**^*a*^

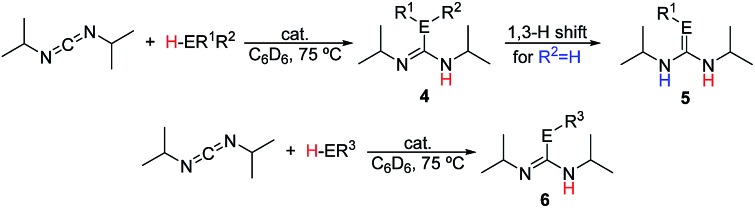					
Entry	Precatalyst	R = (R_2_EH/REH)	Reaction time (h)	Conv^*b*^ (%)	Product
1	**1**	PhNH_2_	6	>99	**5ae**
2	**2**		9	>99	
3	**3**		4	94	
4	**1**	*o*MeO–PhNH_2_	3	>99	**5af**
5	**2**		12	93	
6	**3**		24	96	
7	**1**	*o*Me–PhNH_2_	6	>99	**5ag**
8	**2**		12	93	
9	**3**		6	95	
10	**1**	*p*Cl–PhNH_2_	9	>99	**5ah**
11	**2**		12	>99	
12	**3**		4	>99	
13	**1**	^*i*^PrNH_2_	24	5	**4ai**
14	**3**		24	15	
15	**1**	Et_2_NH	24	23	**4aj**
16	**3**		24	31	
17	**1**	Ph_2_PH	24	58	**4ak**
18	**2**		24	53	
19	**3**		2	>99	
20	**3**	BnSH	2	>99	**6al**
21	**1**	^*t*^BuCCH	24	51	**6am**
22	**2**		24	81	
23	**1**	Me_3_SiCCH	9	96	**6an**
24	**2**		24	96	
25	**3**		12	>99	
26	**1**	PhCCH	9	97	**6ao**
27	**2**		24	97	
28	**3**		18	>99	

^*a*^Reaction conditions: ∼3.5 μmol precatalyst (1 mol%), 600 μL C_6_D_6_, 75 °C.

^*b*^Determined by ^1^H NMR of the crude reaction mixture.

**Fig. 4:**
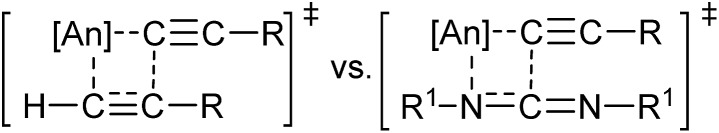
Competitive alkyne and carbodiimide insertion into actinide–acetylide bond.

Encouraged by the diversity of E–H moieties demonstrating favourable reactivity in the actinide-mediated insertion, attention was turned to the possibility of insertion of a C–H bond, a process hitherto unseen in actinide chemistry for heteroatom-containing multiple bonds. Performing this reaction with trimethylsilyl- or phenyl-acetylene generated the desired insertion product with all complexes in excellent yield and short reaction times (Table 1, entries 23–28), providing the substituted amidine. The insertion of 3,3-dimethylbutyne was observed in 51 and 81% conversion after 24 hours after heating in the presence of complexes **1** and **2**, respectively (Table 1, entries 21 and 22), generating *N*,*N*′-diisopropyl-4,4-dimethylpent-2-ynimidamide. The analogous reaction performed with complex **3** produced alkyne dimerization product, however no detectable insertion into the carbodiimide was observed. This finding illustrates the competitive process between the alkyne oligomerisation and insertion into the heterocumulene, revealing a lower barrier energy for insertion into the C≡C bond, favouring the former process (Fig. 4). These reactions were additionally attempted using 1-hexyne, however oligomerisation forming organic enynes was dominant, generating a mixture of products from competitive insertions.

Similar reactions were performed using di-*p*-tolylcarbodiimide (DTC) as the heterocumulene source, and the results are presented in Table 2. Initial analysis of the reactions performed with DTC revealed superb substrate conversions in moderate reaction times. The reaction of aromatic amines with DTC generated the corresponding guanidines in excellent yield after heating at 75 °C (Table 2, entries 1–12). The most apparent trend of these reactions is the shorter reaction times when utilising the thorium metallacycle (**3**), and the observed acceleration of the reaction rate as a more electron deficient aniline is used (Table 2, entries 3, 6, 9, and 12). This trend in reactivity is an expected corollary of the decreased electron donation from the aniline derivatives to the metal centre, resulting in a weaker, more labile bond, and allowing for a more rapid insertion of the carbodiimide into the Th–N bond. Similar short reaction times are observed for the insertion of Ph_2_PH using the thorium precatalyst (**3**) as compared to the uranium precatalysts (**1** and **2**) (Table 2, entries 13–15).

**Table 2:** Catalytic reactions of di-*p*-tolylcarbodiimide with E–H species mediated by complexes **1–3**^*a*^

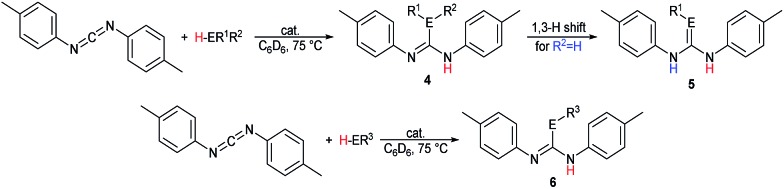					
Entry	Precatalyst	R = (R_2_EH/REH)	Reaction time (h)	Conv^*b*^ (%)	Product
1	**1**	PhNH_2_	24	89	**5be**
2	**2**		24	93	
3	**3**		6	>99	
4	**1**	*o*MeO–PhNH_2_	24	91	**4bf**
5	**2**		24	>99	
6	**3**		24	90	
7	**1**	*o*Me–PhNH_2_	24	96	**4bg**
8	**2**		24	>99	
9	**3**		9	>99	
10	**1**	*p*Cl–PhNH_2_	24	98	**5bh**
11	**2**		24	94	
12	**3**		6	>99	
13	**1**	Ph_2_PH	12	98	**4bk**
14	**2**		24	85	
15	**3**		24	83	
16	**1**	BnSH	6	>99	**6bl**
17	**3**		3	>99	

^*a*^Reaction conditions: ∼3.5 μmol precatalyst (1 mol%), 600 μL C_6_D_6_, 75 °C.

^*b*^Determined by ^1^H NMR of the crude reaction mixture.

Performing these reactions with benzylthiol resulted in rapid insertion into DTC for complexes **1** and **3** (Table 2, entries 16 and 17), however the reaction with precatalyst **2** again resulted in the formation of an intractable green precipitate, which formed immediately upon addition of the thiol. This is most likely due to the formation of insoluble U(iv) dimers bridged by the thiol moiety, which has been previously shown to occur, especially for U(iv) metallocene systems.^57^ Similar reactions were performed with aliphatic amines and terminal alkynes, however no catalytic turnover was observed in any of these reactions, showing that the insertion of the electron deficient carbodiimide is unable to surpass the bonding energy of the actinide–nitrogen and actinide–carbon bonds described.

The scope of these reactions illustrating the robust nature of these complexes was expanded further yet in the insertion into isocyanates and isothiocyanates. Using phenylisocyanate and phenylisothiocyanate as the heterocumulene source, the insertion of diphenylphosphine, benzylthiol, and terminal alkynes was studied, and the results presented in Table 3. It is most notable that the catalytic insertion into these substrates was only achieved using the uranium complexes; this observation is justified by the greater bond-disruption enthalpy of the Th–O and Th–S bond as compared to the uranium analogues (Th–O = 208.0, U–O = 181.0, Th–S = 145.2, and U–S = 121.9 kcal mol^–1^).^58^ These bonding energies indicate a higher barrier of insertion into the Th–S or Th–O species, which is not exceeded under the given reaction conditions, additionally supporting the observed reactivity of the thorium complex with *ortho*-anisidine. The insertion of diphenylphosphine gives only moderate conversions using either uranium complex with both heterocumulenes (Table 3, entries 1, 2, 5, and 6), and a similar trend is observed using benzylthiol, however the reaction of this E–H moiety with phenylisocyanate mediated by the uranium(iii) complex (**1**) shows an enhanced reactivity as compared to the uranium(iv) complex (**2**), providing a 99% conversion to product **8cl** after 24 hours at 75 °C (Table 3, entry 3).

**Table 3:** Catalytic reactions of phenylisocyanate or phenylisothiocyanate with E–H species mediated by complexes **1** and **2**^*a*^

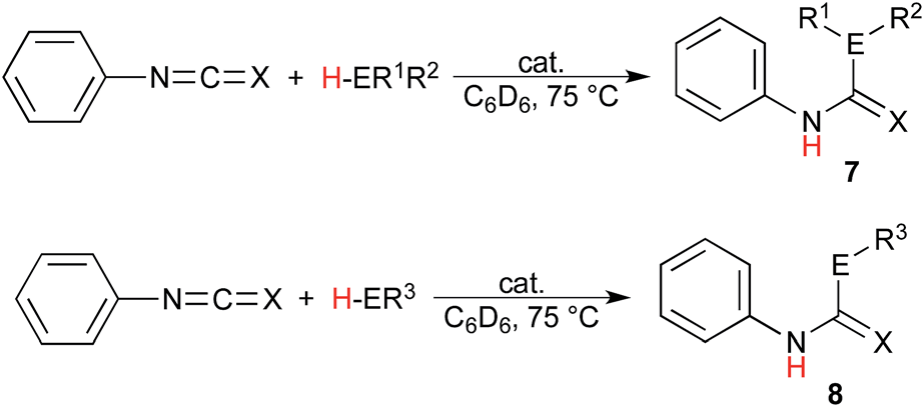					
Entry	Precatalyst	Ph–N = C=X	R = (R_2_EH/REH)	Conv^*b*^ (%)	Product
1	**1**	O	Ph_2_PH	52	**7ck**
2	**2**			64	
3	**1**		BnSH	99	**8cl**
4	**2**			68	
5	**1**	S	Ph_2_PH	49	**7ck**
6	**2**			55	
7	**1**		BnSH	47	**8dk**
8	**2**			52	

^*a*^Reaction conditions: ∼3.5 μmol precatalyst (1 mol%), 600 μL C_6_D_6_, 75 °C, 24 h.

^*b*^Determined by ^1^H NMR of the crude reaction mixture.

**Fig. 5:**
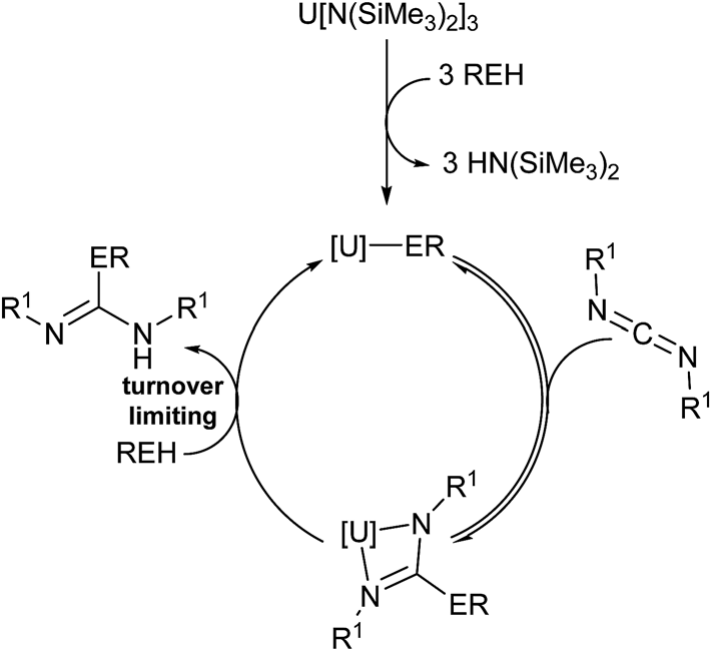
Plausible mechanism of the actinide-mediated catalytic insertion of E–H bond into carbodiimide.

Analysis of the rate order reveals first order kinetics in precatalyst, heterocumulene, and REH, giving rise to the rate eqn (1):
1

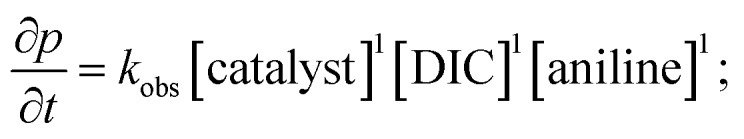

(a more thorough presentation of the kinetic rate law is presented within the ESI†). The mechanism of activation was studied using DIC and aniline. The first step is a rapid protolytic cleavage by the aniline which displaces all hexamethyldisilazide moieties at room temperature (Fig. 5, step (a)), followed by rapid reversible migratory insertion of DIC into the An–N bond (step (b)). A slow protolytic cleavage by an additional equivalent of amine (step (c)) is operative (turnover limiting step) to complete the catalytic cycle with concomitant liberation of the organic product and regeneration of the active catalyst. Deuterium labelling studies were performed in this reaction using aniline-*N*,*N*-d_2_, revealing a primary kinetic isotope effect (*k*_H_/*k*_D_ = 1.22), further supporting the protonolysis of the guanidinate is the turnover limiting step of the catalytic cycle (Fig. 5, step (c)).

Thermodynamic parameters were experimentally calculated from ^1^H NMR data of aniline insertion into DIC using Eyring plots (see ESI†), and the enthalpy of activation (Δ*H*^‡^) for complexes **1–3** were found to be 9.9(1), 10.5(1), and 9.7(1) kcal mol^–1^, respectively, showing a low energetic barrier to the actinide-mediated insertion. The proposed σ-bond metathesis by aniline *via* a four-centred transition state is additionally supported by the remarkably low entropies of activation (Δ*S*^‡^) of –49.5(3), –48.0(4), and –52.8(3) e.u., respectively, for complexes **1–3**. Calculating the Gibbs energy of activation (Δ*G*^‡^) for these reactions at 75 °C showed extremely similar values for each of the precatalysts employed (27.1(1), 27.2(1), and 28.1(1) kcal mol^–1^ for **1–3**, respectively).

## Conclusions

This work presents a series of simple coordinative unsaturated amido–actinide complexes capable of performing the catalytic insertion of E–H bonds into heterocumulenes, where E = C, N, P, or S. The insertion into carbodiimides showed excellent reaction rates and functional group tolerances. The insertion of terminal alkynes represents a new facet of actinide-mediated catalysis. The insertion into phenylisocyanate and phenylisothiocyanate showed a more limited reactivity, however the reaction performed with phosphines and thiols yielded the desired phosphanecarboxamide and carbamothioate.

Deuterium-labelling studies in the reaction of PhND_2_ with DIC revealed a KIE of 1.22, indicating protolytic cleavage of the guanidinate to be the rate-determining step of the catalytic cycle. Preliminary studies in our group show the possibility of using group 4 metal complexes to effect similar chemical transformations. The investigation of further catalytic transformation using these actinide precatalysts is currently underway.

## Supplementary Material

Supplementary information
