# Supplementary material for: Catalytic insertion of E–H bonds (E = C, N, P, S) into heterocumulenes by amido–actinide complexes
Source: Chem Sci. 2015 Oct 29;7(2):939–44. doi: 10.1039/c5sc02746b (PMC5954617; doi:10.1039/c5sc02746b)
Supplement: Supplementary file 1 [file SC-007-C5SC02746B-s001.pdf]

Electronic Supplementary Material (ESI) for Chemical Science.
This journal is © The Royal Society of Chemistry 2015

Supporting Information

Catalytic insertion of E-H bonds (E=C, N, P, S) into heterocumulenes by amido-actinide complexes

Rami J. Batrice and Moris S. Eisen
Schulich Faculty of Chemistry, Technion – Israel Institute of Technology,
Technion City, 32000, Haifa, Israel

Materials and Methods All manipulations of air-sensitive materials were performed with the rigorous exclusion of oxygen and moisture in flamed Schlenk-type glassware or J-Young Teflon valve-sealed NMR tubes on a dual manifold Schlenk line interfaced to a high vacuum (10^{-5} Torr) line, or in a nitrogen-filled Innovative Technologies glovebox with a medium-capacity recirculator (1 – 2 ppm of O2). Argon and nitrogen were purified by passage through MnO oxygen-removal column and a Davison 4 Å molecular sieve column. Hydrocarbon solvents benzene-d_6 (Cambridge Isotopes), toluene (Bio-Lab), and diethyl ether (BioLab) were distilled under vacuum from Na/K alloy, or purified by passage through an activated alumina column under nitrogen atmosphere. Aniline (Sigma Aldrich) was refluxed over stannous chloride and distilled under vacuum, followed by refluxing in calcium hydride under nitrogenous atmosphere and distilling under vacuum. 1,3-di*iso*propylcarbodiimide, phenylisocyanate, phenylisothiocyanate, *ortho*-toluidine, *ortho*-anisidine, *iso*propylamine, diethylamine, and benzyl mercaptan (Sigma Aldrich) were distilled from sodium bicarbonate under nitrogen atmosphere. 1,3-di-*p*-tolylcarbodiimide and *para*-chloroaniline were dried under vacuum (10^{-6}) for 12 hours on a high vacuum line. 3,3-dimethylbutyne, trimethylsilylacetylene, and phenylacetylene (ABCR) were distilled under vacuum and degassed by three freeze-pump-thaw cycles. Diphenylphosphine (Sigma Aldrich) was inserted into the glovebox and used as received. All the aforementioned reagents were stored in an inert atmosphere glovebox prior to use. The actinide complexes $U[N(SiMe_3)_2]_3$ (**1**)[1] and $[(Me_3Si)_2N]_2An[\kappa^2\text{-}(N,C)\text{-}CH_2Si(CH_3)_2N(SiMe_3)]$ (An = U (**2**),[2] Th (**3**)[3]) were prepared according to published methods.

NMR spectra were recorded on Bruker Avance 300, Bruker Avance III 400, or Bruker Avance 500 spectrometers on crude reaction mixtures. Chemical shifts for ^{1}H- and ^{13}C-NMR are referenced to internal protiosolvent and reported relative to tetramethylsilane. *J*-values are reported for ^{1}H NMR coupling constants in the unit of Hertz (Hz). Known products were compared to previously reported data.

Synthesis of $PhND_2$

Freshly distilled aniline (10.2 g, 109.5 mmol) was stirred vigorously with deuterium oxide (20 g, 1 mol) for three hours then loaded into a separatory funnel. The aqueous layer was removed, and the aniline washed with three 10 mL portions of deuterium oxide. The aniline was dried over BaO, distilled under vacuum, and stored over 4Å molecular sieves in an inert atmosphere glovebox prior to use. ^{1}H NMR analysis shows >98% deuteration.

Synthesis of Me_3SiCCD

Trimethylsilylacetylene (10 mL, 70 mmol) was syringed into a thick-walled Schlenk tube containing a 1.6 M solution of ^{n}BuLi in hexane (37 mL, 59 mmol) at -95 °C. The mixture was allowed to warm slowly to 0 °C and stirred for 30 minutes. The temperature was then allowed to rise to room temperature, and the volatiles removed in vacuo to yield a white solid. The flask was cooled to -85 °C, and under nitrogen flush, an excess of D2O (20 mL) was slowly added by syringe. The tube was sealed and slowly warmed to 0 °C and the mixture stirred vigorously for 10 minutes until all solids dissolved. The mixture was

separated, and the organic layer distilled under nitrogen. The distillate was run through a plug of MgSO4, redistilled, and stored over 4 Å molecular sieves, yielding 8 mL of Me3SiC≡CD. ^{1}H NMR analysis shows >99% deuteration.

General procedure for actinide-mediated insertion of nucleophiles with heterocumulenes
A sealable J. Young NMR tube was loaded with 2.5 mg (3.5 μmol) of the respective actinide catalyst (1-3) from a C_6D_6 stock solution inside the glovebox. The respective heterocumulene (350 μmol, 100 equiv.) and nucleophile (350 μmol, 100 equiv.), then immediately sealed and heated to 75 °C. The progress of the reactions were monitored by ^{1}H NMR spectroscopy. The products were identified by according to ^{1}H, ^{13}C, and 2D NMR spectroscopy (where necessary), as well as MS analysis. Where applicable, product data were compared to previously reported spectra.

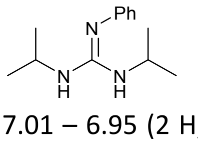

1,3-diisopropyl-2-phenylguanidine (5ae):[4] The insertion of aniline (32 μL, 350 μmol) and 1,3-diisopropylcarbodiimide (55 μL, 350 μmol) was carried out following the general procedure described above. δ $_H$ (300 MHz, C_6D_6) 7.24 – 7.15 (2 H, m, *m*-Ar*H*), 7.01 – 6.95 (2 H, m, *o*-Ar*H*), 6.90 – 6.82 (1 H, m, *p*-Ar*H*), 3.67 (2 H, hept, *J* 6.4, C*H*(CH$_3$)$_2$), 3.45 (2 H, bs, N*H*), 0.95 (12 H, d, *J* 6.4, CH(C*H$_3$*)$_2$). MS (APCI): m/z 220.1864 (M + H)*

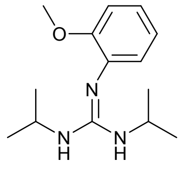

1,3-diisopropyl-2-(2-methoxyphenyl)guanidine (5af):[4] The insertion of *o*-anisidine (39 μL, 350 μmol) and 1,3-diisopropylcarbodiimide (55 μL, 350 μmol) was carried out following the general procedure described above. δ $_H$ (300 MHz, C_6D_6) 7.13 (1 H, dt, *J* 8.1, 4.2, *p*-Ar*H*), 7.00 – 6.86 (2 H, m, *o/m*-Ar*H*), 6.76 (1 H, dd, *J* 7.6, 1.7, *m*-Ar*H*), 3.79 – 3.64 (2 H, m, C*H*(CH$_3$)$_2$), 3.50 (2 H, bs, N*H*), 3.46 (3 H, s, Ar-OC*H$_3$*), 0.94 (12 H, d, *J* 6.4, CH(C*H$_3$*)$_2$). MS (APCI): m/z 250.1971 (M + H)*

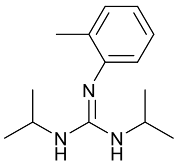

1,3-diisopropyl-2-(o-tolyl)guanidine (5ag):[4] The insertion of *o*-toluidine (38 μL, 350 μmol) and 1,3-diisopropylcarbodiimide (55 μL, 350 μmol) was carried out following the general procedure described above. δ $_H$ (300 MHz, C_6D_6) 7.31 – 7.19 (2 H, m, *m*-Ar*H*), 7.08 (1 H, dd, *J* 7.8, 1.3, *p*-Ar*H*), 7.02 – 6.95 (1 H, m, *o*-Ar*H*), 3.68 (2 H, bs, C*H*(CH$_3$)$_2$), 3.41 (2 H, bs, N*H*), 2.40 (3 H, s, Ar-C*H$_3$*), 0.95 (12 H, d, *J* 6.4 CH(C*H$_3$*)$_2$). MS (APCI): m/z 234.2015 (M + H)*

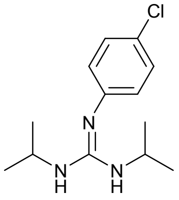

2-(4-chlorophenyl)-1,3-diisopropylguanidine (5ah):[4] The insertion of *p*-chloroaniline (45 mg, 350 μmol) and 1,3-diisopropylcarbodiimide (55 μL, 350 μmol) was carried out following the general procedure described above. δ $_H$ (300 MHz, C_6D_6) 7.17 (2 H, d, *J* 8.6, *m*-Ar*H*), 6.85 (2 H, d, *J* 8.6, *o*-Ar*H*), 3.66 – 3.51 (2 H, m, C*H*(CH$_3$)$_2$), 3.43 (2 H, bs, N*H*), 0.87 (12 H, d, *J* 6.3, CH(C*H$_3$*)$_2$). MS (APCI): [M + H]* *obsd.* m/z 254.1486, *calcd. for* $C_{13}H_{21}ClN_3$ 254.1424.

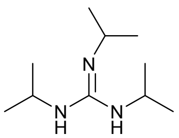

1,2,3-triisopropylguanidine (4ai):[4] The insertion of *iso*propylamine (29 μL, 350 μmol) and 1,3-diisopropylcarbodiimide (55 μL, 350 μmol) was carried out following the general procedure described above. δ $_H$ (300 MHz, C_6D_6) 3.41 – 3.25 (3 H, m, C*H*(CH$_3$)$_2$), 3.16 (2 H, bs, N*H*), 1.06 – 1.01 (18 H, d, CH(C*H$_3$*)$_2$). MS (APCI): m/z 186.2020 (M + H)*

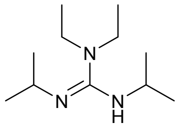

1,1-diethyl-2,3-diisopropylguanidine (4aj):[4] The insertion of diethylamine (36 µL, 350 µmol) and 1,3-diisopropylcarbodiimide (55 µL, 350 µmol) was carried out following the general procedure described above. δ_H (300 MHz, C_6D_6) 3.44 – 3.25 (2 H, m, C*H*(CH_3)$_2$), 3.12 (4 H, q, *J* 7.1, NC*H*$_2$$CH_3$), 3.01 (1 H, bs, N*H*), 1.20 (6 H, d, *J* 6.4, CH(C*H*$_3$)$_2$), 0.98 (6 H, t, *J* 7.1, CH(C*H*$_3$)$_2$), 0.92 (6 H, d, *J* 6.4, NCH_2C*H*$_3$). MS (ESI): m/z 200.1741 (M + H)*

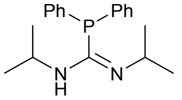

***N,N'*-diisopropyl-1,1-diphenylphosphanecarboximidamide (4ak):**[4] The insertion of diphenylphosphine (61 µL, 350 µmol) and 1,3-diisopropylcarbodiimide (55 µL, 350 µmol) was carried out following the general procedure described above. δ_H (300 MHz, C_6D_6) 7.47 – 7.40 (4 H, m, *m*-Ar*H*), 7.38 – 7.32 (2 H, m, *p*-Ar*H*), 7.11 – 7.01 (4 H, m, *o*-Ar*H*), 4.44 – 4.19 (2 H, m, C*H*(CH_3)$_2$), 3.62 (1 H, bs, N*H*), 1.23 – 1.17 (6 H, m CH(C*H*$_3$)$_2$), 0.94 (6 H, d, *J* 6.4, CH(C*H*$_3$)$_2$). MS (APCI): m/z 313.1951 (M + H)*

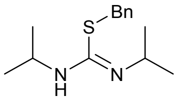

Benzyl-*N,N'*-diisopropylcarbamimidothioate (6al):[4] The insertion of benzyl mercaptan (41 µL, 350 µmol) and 1,3-diisopropylcarbodiimide (55 µL, 350 µmol) was carried out following the general procedure described above. δ_H (300 MHz, C_6D_6) 7.11 – 6.66 (5 H, m, Ar*H*), 3.84 (2 H, m, C*H*(CH_3)$_2$), 3.43 (2 H, s, C*H*$_2$Ar), 3.06 (1 H, bs, N*H*), 0.87 (12 H, m, CH(C*H*$_3$)$_2$). MS (APCI): m/z 251.1639 (M + H)*

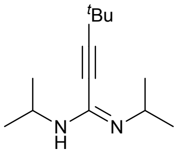

***N,N'*-diisopropyl-4,4-dimethylpent-2-ynimidamide (6am):**[5] The insertion of 3,3-dimethylbutyne (43 µL, 350 µmol) and 1,3-diisopropylcarbodiimide (55 µL, 350 µmol) was carried out following the general procedure described above. δ_H (300 MHz, C_6D_6) 4.13 (1 H, m), 3.74 (1 H, bs), 3.34 (1 H, hept, *J* 6.4), 1.13 (9 H, s), 1.04 (12 H, d, *J* 6.4). δ_C (126 MHz, C_6D_6) 144.02, 99.55, 71.57, 52.67, 46.40, 30.70, 30.51, 27.88, 27.37.HRMS (ESI): [M + H]* *obsd.* m/z 209.2010, *calcd. for* $C_{13}H_{25}N_2$ 209.2018.

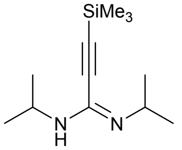

***N,N'*-diisopropyl-3-(trimethylsilyl)propiolimidamide (6an):** The insertion of trimethylsilylacetylene (50 µL, 350 µmol) and 1,3-diisopropylcarbodiimide (55 µL, 350 µmol) was carried out following the general procedure described above. δ_H (300 MHz, C_6D_6) 4.23 – 4.08 (1 H, m, C*H*(CH_3)$_2$), 3.77 (1 H, s, N*H*), 3.34 (1 H, hept, *J* 6.3, C*H*(CH_3)$_2$), 1.04 (12 H, d, *J* 6.4, CH(C*H*$_3$)$_2$), 0.14 (9 H, s, Si(C*H*$_3$)$_3$). δ_C (126 MHz, C_6D_6) 140.15 (*ipso-C*), 95.93 (C≡*C*-Si(CH_3)$_3$), 89.71 (*C*≡C-Si(CH_3)$_3$), 46.49 (*C*H(CH_3)$_2$), 24.82 (CH(*C*H$_3$)$_2$), 2.69 (Si(*C*H$_3$)$_3$). HRMS (ESI): m/z 225.1768 (M + H)*

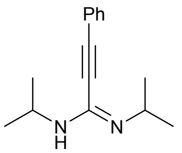

***N,N'*-diisopropyl-3-phenylpropiolimidamide (6ao):**[6] The insertion of phenylacetylene (38 µL, 350 µmol) and 1,3-diisopropylcarbodiimide (55 µL, 350 µmol) was carried out following the general procedure described above. δ_H (300 MHz, C_6D_6) 7.44 – 7.33 (2 H, m, *o*-Ar*H*), 7.01 – 6.89 (3 H, m, *m/p*-Ar*H*), 4.31 – 4.15 (1 H, m, C*H*(CH_3)$_2$), 3.85 (1 H, bs, N*H*), 3.41 – 3.26 (1 H, m, C*H*(CH_3)$_2$), 1.55 – 0.81 (12 H, m, CH(C*H*$_3$)$_2$). MS (APCI): m/z 229.1757 (M + H)*

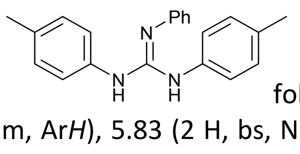

2-phenyl-1,3-di-p-tolylguanidine (5be): The insertion of aniline (32 µL, 350 µmol) and 1,3-di-*p*-tolylcarbodiimide (55 µL, 350 µmol) was carried out following the general procedure described above. δ_H (300 MHz, C_6D_6) 6.93 (13 H, m, Ar*H*), 5.83 (2 H, bs, N*H*), 2.11 (6 H, s, *p*-ArC*H*$_3$). δ_C (126 MHz, C_6D_6) 145.13 (*ipso-C*), 144.91 (Ar *C-N*), 144.67 (Ar *C*-N), 130.12 (Ar *C*-CH_3), 129.90(Ar *C*-H), 129.52(Ar *C*-H), 128.35 (Ar *C*-H), 124.50 (Ar *C*-H), 121.47 (Ar *C*-H), 20.77 (Ar C-*C*H$_3$). HRMS (ESI): m/z 316.1819 (M + H)*

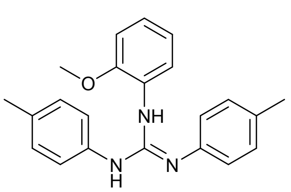

2-(2-methoxyphenyl)-1,3-di-p-tolylguanidine (4bf): The insertion of *o*-anisidine (39 µL, 350 µmol) and 1,3-di-*p*-tolylcarbodiimide (55 µL, 350 µmol) was carried out following the general procedure described above. δ $_H$ (300 MHz, C_6D_6) 9.04 (2 H, s, N*H*), 7.54 – 6.16 (12 H, m, Ar*H*), 3.09 (3 H, s, OCH_3), 2.09 (6 H, s, *p*-ArCH_3). δ $_C$ (126 MHz, C_6D_6) 145.91 (*ipso-C*), 145.83 (Ar *C-N*), 145.60 (Ar *C*-O), 145.02 (Ar *C*-N), 144.86 (Ar *C*-N), 137.03 (Ar *C*-CH_3), 130.24 (Ar *C*-CH_3), 130.11 (Ar C-H), 129.90 (Ar C-H), 124.87 (Ar C-H), 122.89 (Ar C-H), 121.64 (Ar C-H), 120.45 (Ar C-H), 110.74 (Ar C-H), 109.46 (Ar C-H), 55.22 (O-CH_3), 20.93 (CH_3), 20.78 (CH_3). HRMS (ESI): m/z 346.1910 (M + H)*

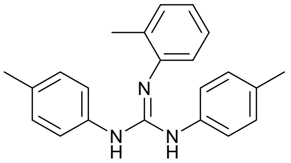

2-(o-tolyl)-1,3-di-p-tolylguanidine (4bg): The insertion of *o*-toluidine (38 µL, 350 µmol) and 1,3-di-*p*-tolylcarbodiimide (55 µL, 350 µmol) was carried out following the general procedure described above. δ $_H$ (300 MHz, C_6D_6) 7.79 – 6.26 (12 H, m, Ar*H*), 5.77 (2 H, s, N*H*), 2.08 (6 H, s, *p*-ArCH_3), 1.81 (3 H, s, *o*-ArCH_3). δ $_C$ (126 MHz, C_6D_6) 145.60 (*ipso-C*), 145.07 (Ar *C-N*), 144.48 (Ar *C*-N), 142.80 (Ar *C*-N), 137.03 (Ar *C*-H), 131.10 (Ar *C*-CH_3), 130.10 (Ar *C*-CH_3), 130.03 (Ar *C*-CH_3), 129.46 (Ar *C*-H), 129.06 (Ar *C*-H), 128.35 (Ar *C*-H), 127.74 (Ar *C*-H), 127.33 (Ar *C*-H), 127.31 (Ar *C*-H), 118.62 (Ar *C*-H), 20.93 (C-CH_3), 20.78 (C-CH_3), 17.30 (C-CH_3). HRMS (ESI): m/z 330.1961 (M + H)*

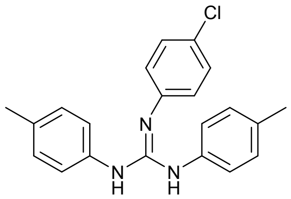

2-(4-chlorophenyl)-1,3-di-p-tolylguanidine (5bh): The insertion of *p*-chloroaniline (45 mg, 350 µmol) and 1,3-di-*p*-tolylcarbodiimide (55 µL, 350 µmol) was carried out following the general procedure described above. δ $_H$ (300 MHz, C_6D_6) 7.34 – 6.60 (12 H, m, Ar*H*), 5.74 (2 H, s, N*H*), 2.07 (6 H, s, *p*-ArCH_3). δ $_C$ (126 MHz, C_6D_6) 145.66 (*ipso-C*), 145.25 (Ar *C*-N), 130.17 (Ar *C*-N), 129.46 (Ar *C*-N), 129.28 (Ar *C*-Cl), 128.35 (Ar *C*-H), 122.82 (Ar *C*-CH_3), 122.66 (Ar *C*-CH_3), 116.14 (Ar *C*-H), 20.77 (C-CH_3). HRMS (ESI): m/z 350.1420 (M + H)*

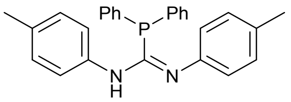

1,1-diphenyl-N,N'-di-p-tolylphosphanecarboximidamide (4bk):[7] The insertion of diphenylphosphine (61 mg, 350 µmol) and 1,3-di-*p*-tolylcarbodiimide (55 µL, 350 µmol) was carried out following the general procedure described above. δ $_H$ (300 MHz, C_6D_6) 7.68 (4 H, d, *J* 8.3, Ar*H*), 7.42 – 7.31 (4 H, m, PAr*H*), 7.08 – 6.86 (6 H, m, PAr*H*), 6.81 (4 H, d, *J* 8.5, Ar*H*), 6.43 (1 H, bs, N*H*), 2.07 (3 H, s, *p*-ArCH_3), 2.05 (3 H, s, *p*-ArCH_3). MS (APCI): m/z 409.1941
(M + H)*

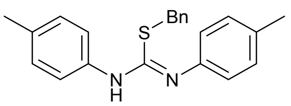

Benzyl-N,N'-di-p-tolylcarbamimidothioate (6bl): The insertion of benzyl mercaptan (41 mg, 350 µmol) and 1,3-di-*p*-tolylcarbodiimide (55 µL, 350 µmol) was carried out following the general procedure described above. δ $_H$ (300 MHz, C_6D_6) 7.32 – 6.71 (13 H, m, Ar*H*), 6.14 (1 H, s, N*H*), 3.26 (2 H, s, CH_2), 2.10 (6 H, s, *p*-ArCH_3). δ $_C$ (101 MHz, C_6D_6) 148.08, 145.11, 141.58, 138.08, 132.60, 129.82, 129.33, 128.84, 128.76, 127.53, 127.08, 121.68, 36.10, 20.89. HRMS (ESI): m/z 347.1580 (M + H)*

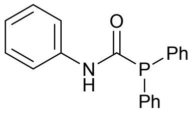

N,1,1-triphenylphosphanecarboxamide (7ck):[8] The insertion of diphenylphosphine (61 mg, 350 µmol) and phenylisocyanate (38 µL, 350 µmol) was carried out following

the general procedure described above. δ $_H$ (300 MHz, C_6D_6) 9.03 (1 H, s, N*H*), 7.74 – 6.72 (15 H, m, Ar*H*). MS (APCI): m/z 306.1014 (M + H)*

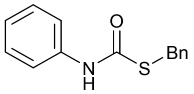

S-benzyl phenylcarbamothioate (8cl):[9] The insertion of benzyl mercaptan (41 mg, 350 µmol) and phenylisocyanate (38 µL, 350 µmol) was carried out following the general procedure described above. δ $_H$ (300 MHz, C_6D_6) 7.42 – 6.55 (10 H, m, Ar*H*), 4.10 (1 H, bs, N*H*), 3.94 (2 H, s, CH_2). MS (APCI): m/z 244.0788 (M + H)*

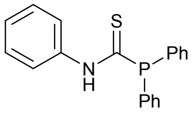

N,1,1-triphenylphosphanecarbothioamide (7ck):[10] The insertion of diphenylphosphine (61 mg, 350 µmol) and phenylisothiocyanate (42 µL, 350 µmol) was carried out following the general procedure described above. δ $_H$ (300 MHz, C_6D_6) 8.70 (1 H, bs, N*H*), 7.73 – 7.25 (6 H, m, Ar*H*), 7.12 – 6.53 (9 H, m, Ar*H*). MS (ESI): m/z 322.0463 (M + H)*

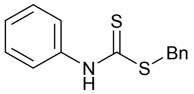

Benzyl phenylcarbamodithioate (8dk):[11] The insertion of benzyl mercaptan (41 mg, 350 µmol) and phenylisothiocyanate (42 µL, 350 µmol) was carried out following the general procedure described above. δ $_H$ (300 MHz, C_6D_6) 8.46 (1 H, bs, N*H*), 7.27 – 6.49 (10 H, m, Ar*H*), 4.53 (2 H, s, CH_2). HRMS (ESI): m/z 260.0570 (M + H)*

Preparative Scale Synthesis of 2-(2-methoxyphenyl)-1,3-di-p-tolylguanidine (4bf): A Schlenk tube equipped with stir bar was loaded with 161 mg 1,3-di-*p*-tolylcarbodiimide (724 µmol), followed by the addition of 10 mL toluene. A stock solution of 6 mg of complex **2** was added to the DTC solution, followed by the addition of 81.8 µL *o*-anisidine (89.2 mg, 724 µmol). The tube was sealed and submerged in a preheated oil bath (75 °C) for 24 hours with stirring. After completion, the reaction mixture was cooled to room temperature and evaporated to dryness under vacuum. The residue was dissolved in dichloromethane, then purified using column chromatography on silica gel using a gradient eluent of 10% ethyl acetate in hexane to pure ethylacetate, yielding 229 mg of a white viscid solid (92 % isolated yield).

δ $_H$ (400 MHz, CD_2Cl_2) 7.89 (1H, bs, N*H*), 7.21 – 7.05 (9H, m, Ar*H*), 7.03 – 6.87 (3 H, m, Ar*H*), 6.16 (1 H, bs, N*H*), 3.79 (3 H, s, OCH_3), 2.36 (3 H, s, *p*-ArCH_3), 2.34 (3 H, s, *p*-ArCH_3). δ $_C$ (101 MHz, CD_2Cl_2) 149.70 (*ipso-C*), 145.96 (Ar *C-N*), 145.67 (Ar *C-N*), 145.45 (Ar *C-N*), 132.96 (Ar *C*-O), 130.24 (Ar *C*-CH_3), 130.14 (Ar *C*-CH_3), 122.70 (Ar C-H), 122.18 (Ar C-H), 121.31 (Ar C-H), 121.28 (Ar C-H), 111.19 (Ar C-H), 110.92 (Ar C-H), 56.08 (O-*C*H_3), 20.93 (*C*H_3), 20.90 (*C*H_3). (**See Figures S1-S2**).

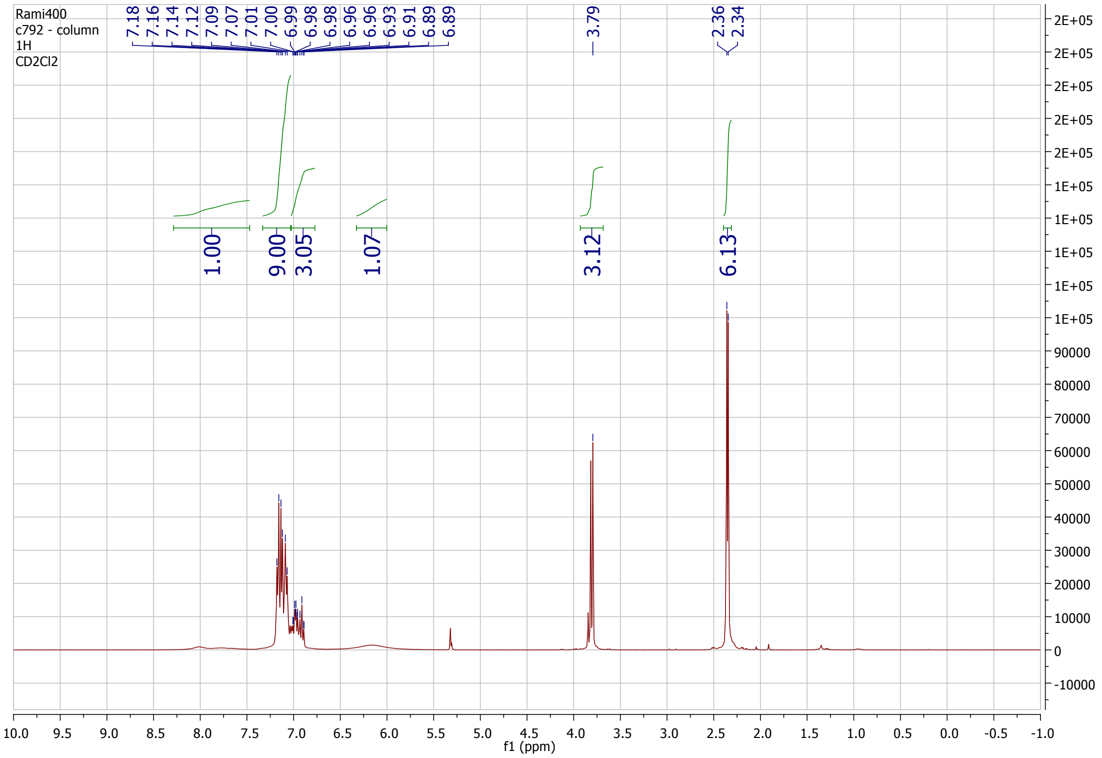

Figure S 1. ^{1}H-NMR of product **4bf** from preparative scale reaction.

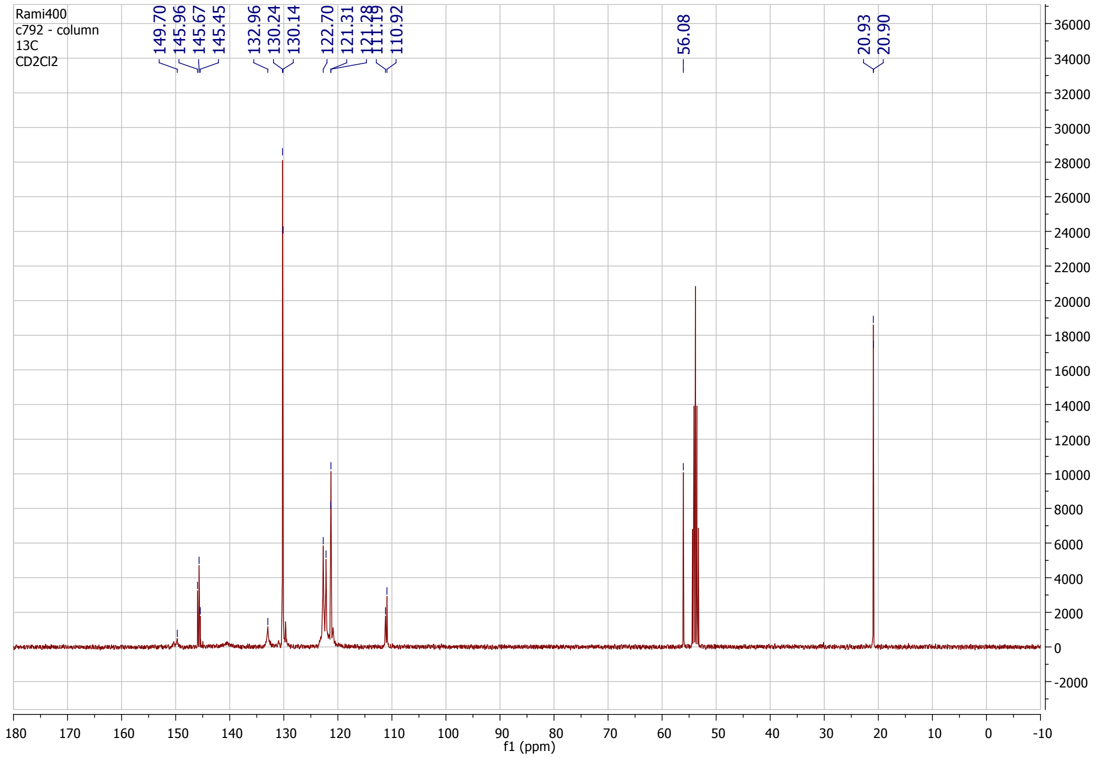

Figure S 2. ^{13}C-NMR of product **4bf** from preparative scale reaction.

Reactivity studies of precatalyst activation

Experiments were prepared using stock solutions of catalysts **1**-**3**. A solution of 10 mg of desired catalyst in 500 µL C_6D_6 was loaded into a Teflon sealed J.-Young NMR tube. The ^{1}H NMR spectrum for each sample was acquired, followed by the addition of either 1,3-diisopropylcarbodiimide (4 equiv.) or aniline (4 equiv.). The samples were shaken and the ^{1}H NMR spectrum again acquired. It was seen for each sample that rapid protonolysis by aniline occurs, evident by the formation of hexamethyldisilazane according to NMR spectroscopy. The NMR spectra of the reactions of complexes **1**-**3** with four equivalents of aniline are shown below (**Figures S3-S5**) at room temperature, as well as the activation studies with four equivalents. When only 3 equivalents of amine are added to complex **1**, there is a precipitate that only dissolved by adding the additional equivalent. with 1,3-diisopropylcarbodiimide at the reaction performed temperature (at room temperature there is no reaction) (**Figure S6-S8**).

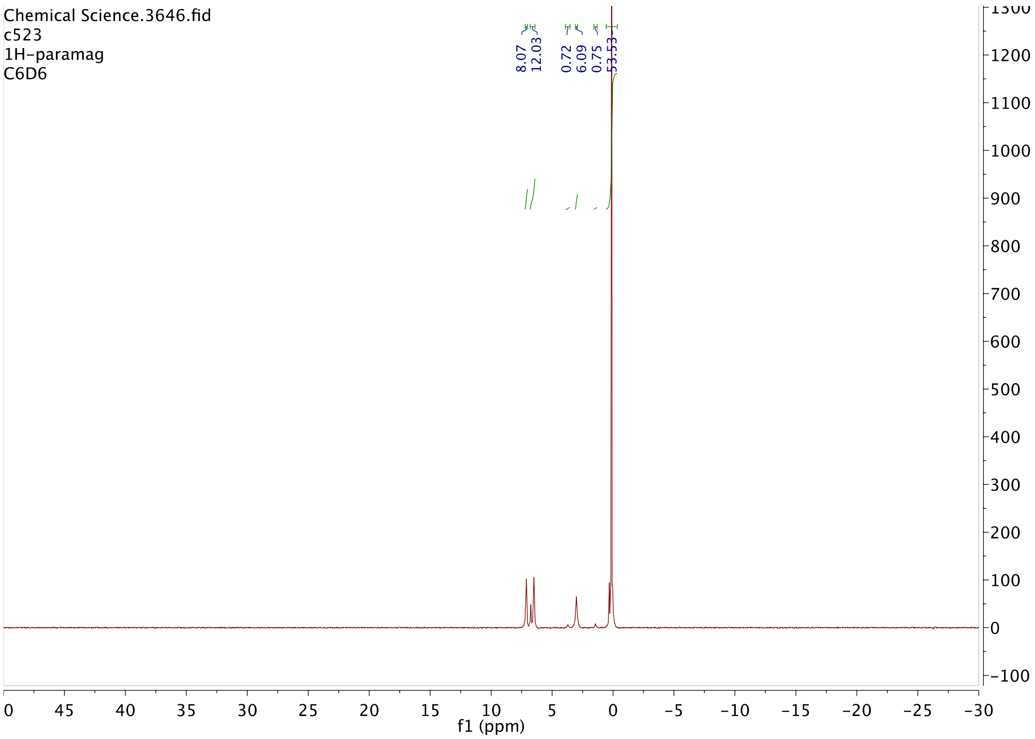

Figure S 3. ^{1}H-NMR of complex **1** after activation with four equivalents of aniline.

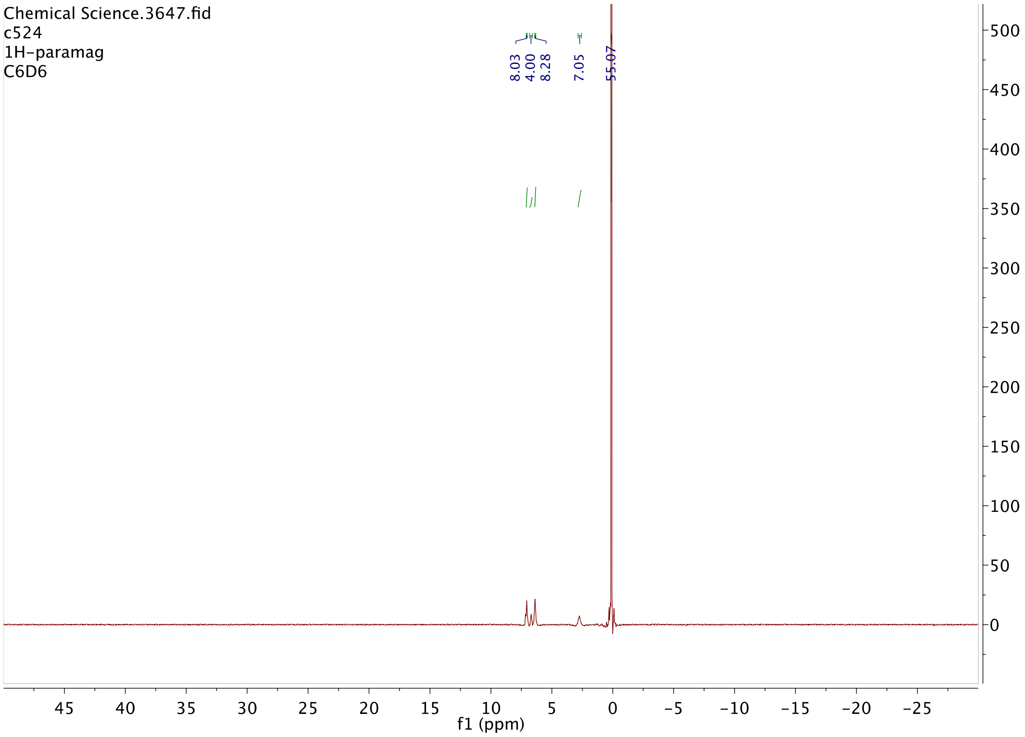

Figure S 4. ^{1}H-NMR of complex **2** after activation with four equivalents of aniline at room temperature.

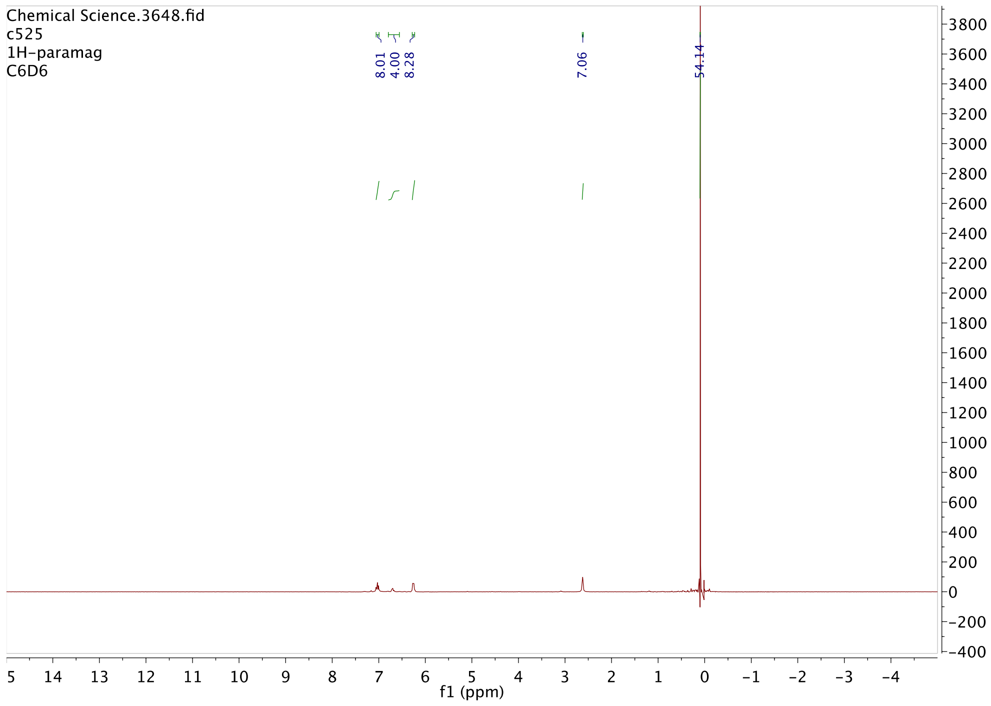

Figure S 5. ^{1}H-NMR of complex **3** after activation with four equivalents of aniline.

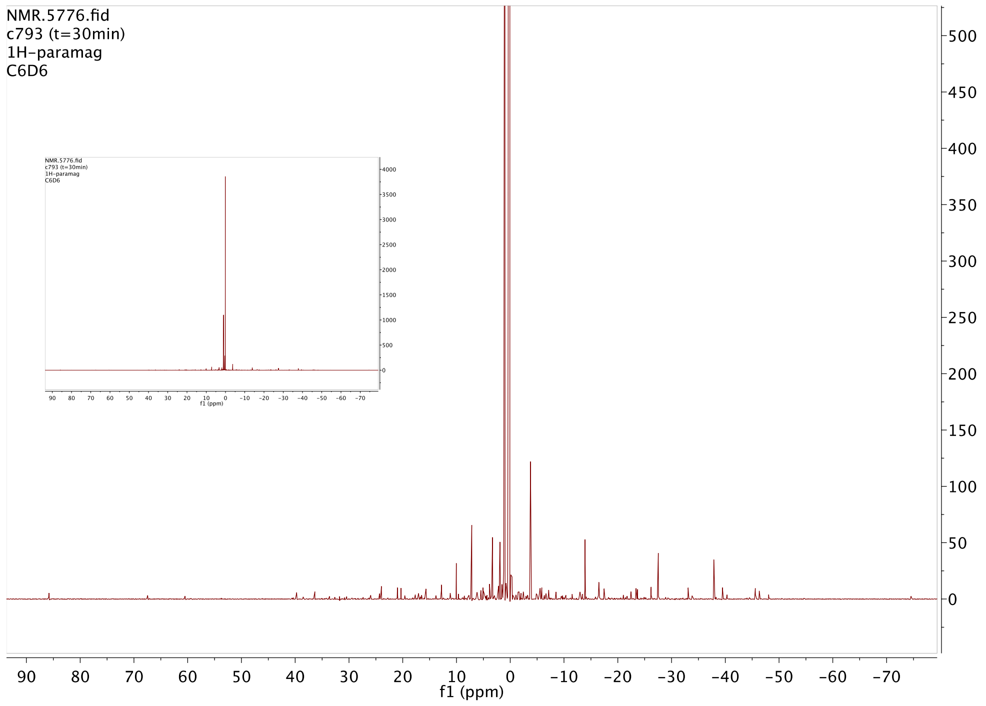

Figure S 6. ^{1}H-NMR of complex **1** after activation with four equivalents of 1,3-diisopropylcarbodiimide.

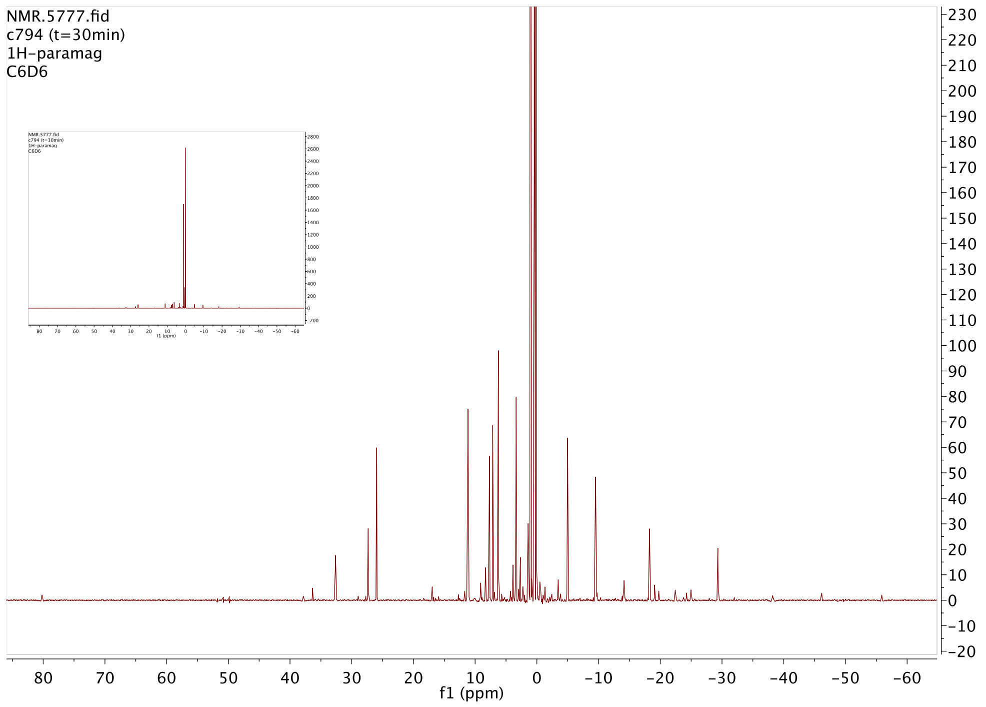

Figure S 7. ^{1}H-NMR of complex **2** after activation with four equivalents of 1,3-diisopropylcarbodiimide.

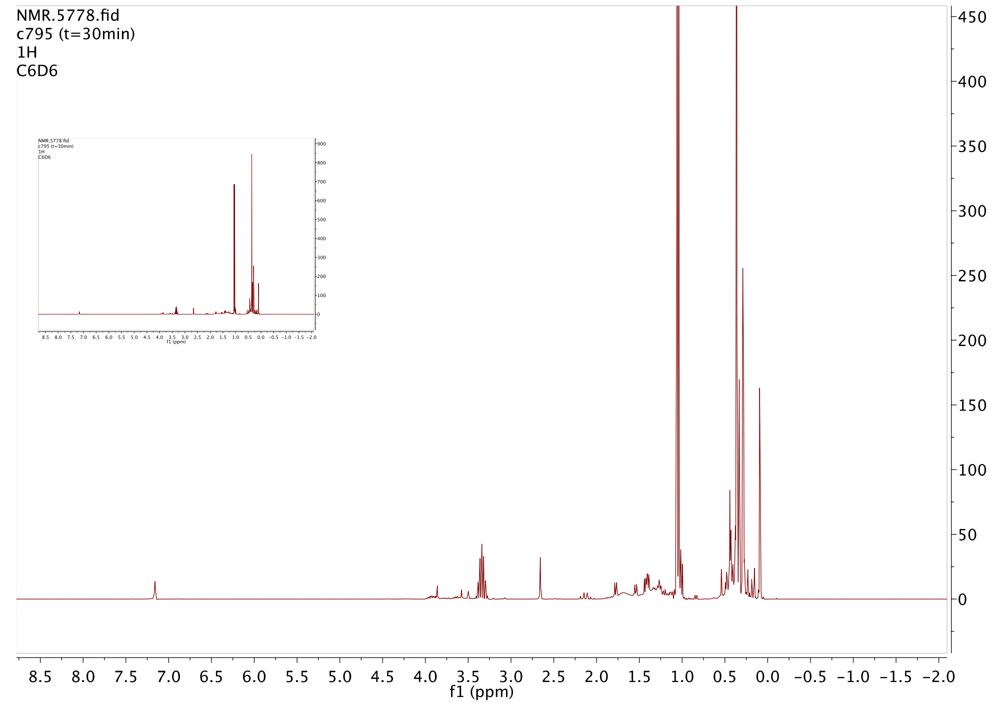

Figure S 8. ^{1}H-NMR of complex **3** after activation with four equivalents of 1,3-diisopropylcarbodiimide.

Kinetic studies of aniline insertion into diisopropylcarbodiimide

In a typical experiment, an NMR sample was prepared as described above. Experiments for reagent-order determination were performed at variable concentrations of catalyst, aniline, and carbodiimide, spanning one order of magnitude concentration differences while keeping the other reagent concentration constant. The sample tube was inserted into the probe of the Bruker Avance 300 spectrometer which had been previously set to the desired temperature (T = 75 ± 0.1 °C; checked with ethylene glycol temperature standard). Data were acquired every five minutes up to one and one-half hours, and product concentrations were measured from the area of the N-H proton of product **5ae**. Reaction rates were determined by least-squares fit of product concentration versus time, and the collective rate data plotted to determine reagent orders. Representative plots are shown in **Figures S9-S11**.

Activation parameters ($\Delta H^{\ddagger}$ and $\Delta S^{\ddagger}$) were calculated from acquired kinetic data using Eyring plots. In a typical experiment, an NMR sample was prepared as described above. The sample tube was inserted into the probe of the Bruker Avance 300 spectrometer which had been previously set to the desired temperature over the range of 35 – 75 °C (T ± 0.1 °C; checked with ethylene glycol temperature standard). Data were acquired five minutes up to one and one-half hours, and product concentrations were measured from the area of the N-H proton of product **5ae**. Reaction rates were determined by least-squares fit of product concentration versus time. Eyring plots were generated in the usual manner, and the $\Delta H^{\ddagger}$ and $\Delta S^{\ddagger}$ values for activation calculated from the slope and intercept of the least-squares fit, respectively. Representative plots are shown in **Figures S12-S14**.

Reaction Order Plots

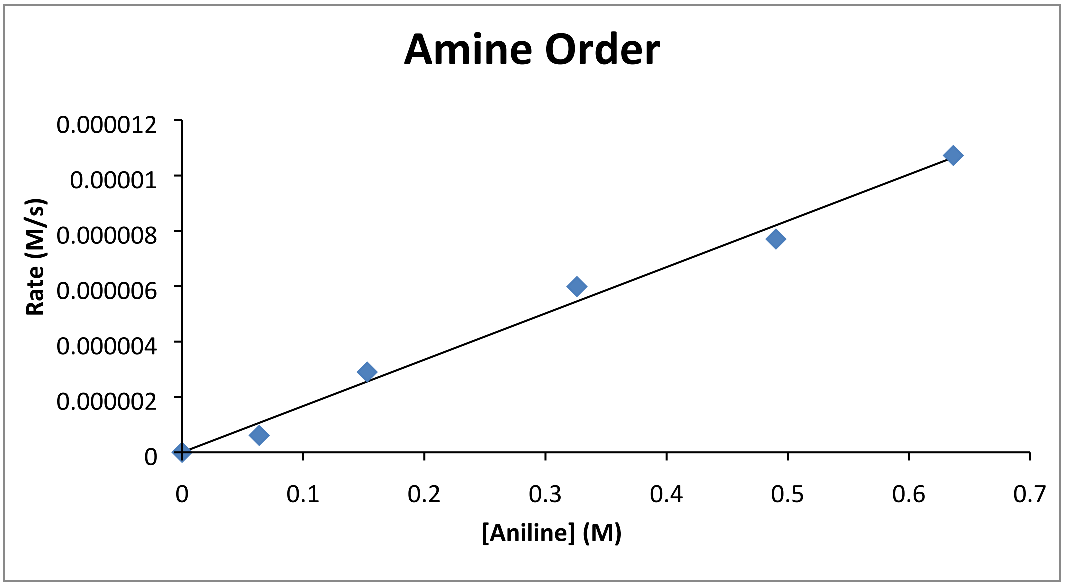

Figure S 9. Plot of aniline concentration (M) versus reaction rate showing first-order kinetics.

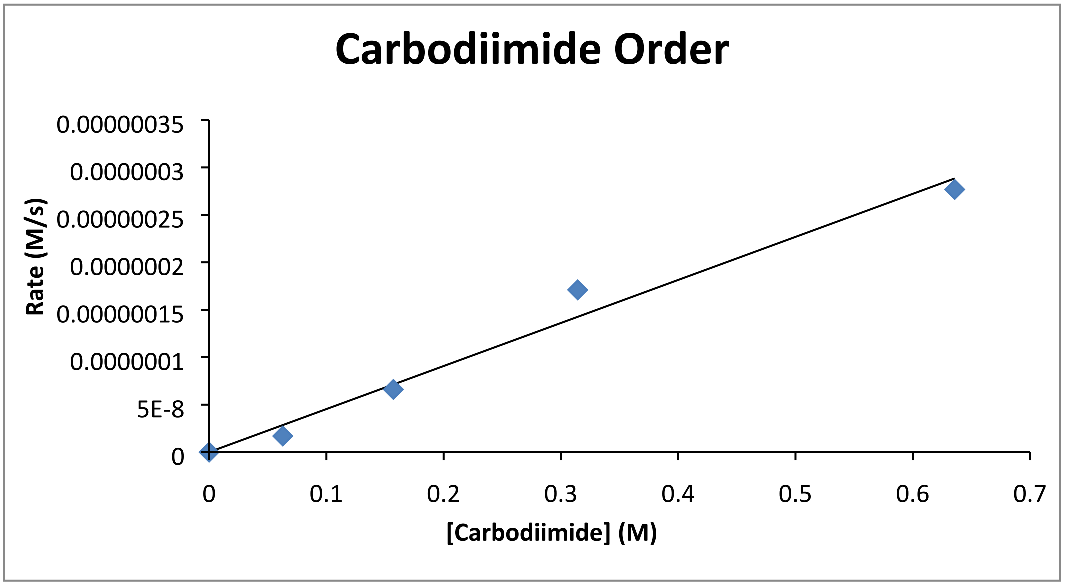

Figure S 10. Plot of diisopropylcarbodiimide concentration (M) versus reaction rate showing first-order kinetics.

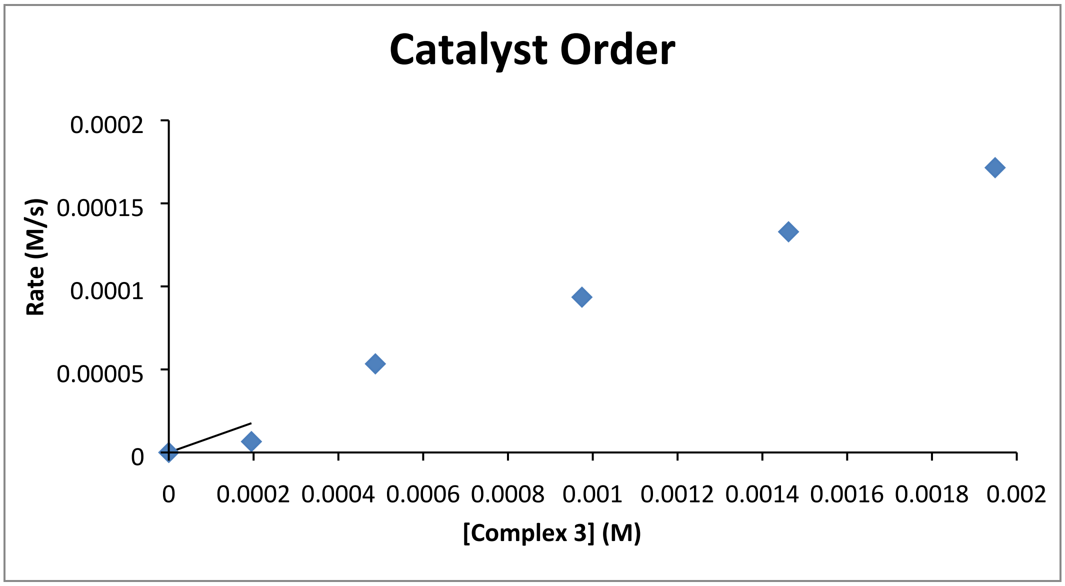

Figure S 11. Plot of catalyst **3** concentration (M) versus reaction rate showing first-order kinetics.

Eyring plots of aniline insertion into 1,3-diisopropylcarbodiimide

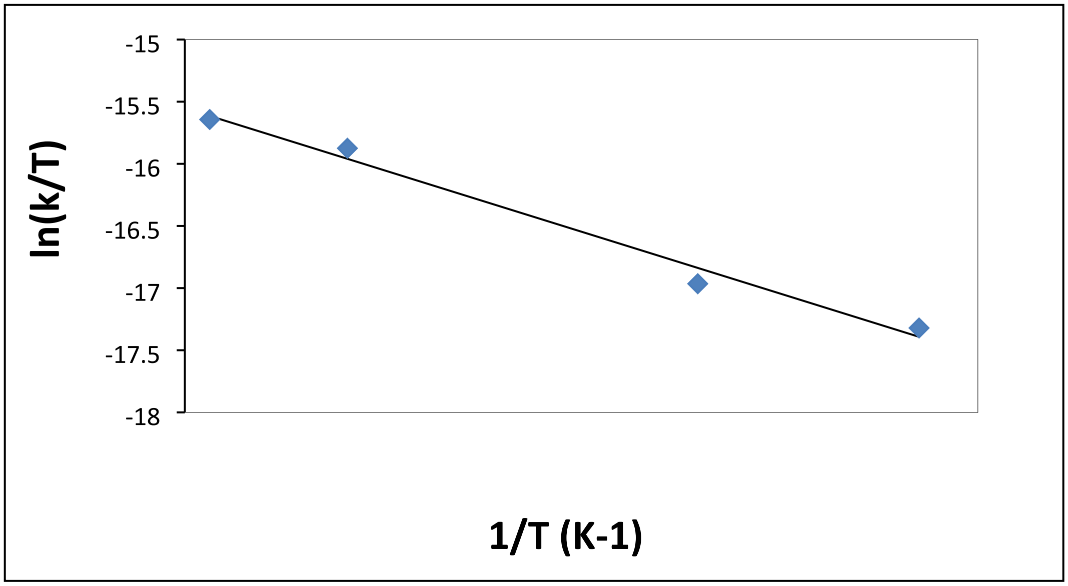

Figure S 12. Eyring plot of aniline insertion into diisopropylcarbodiimide by catalyst **1**.

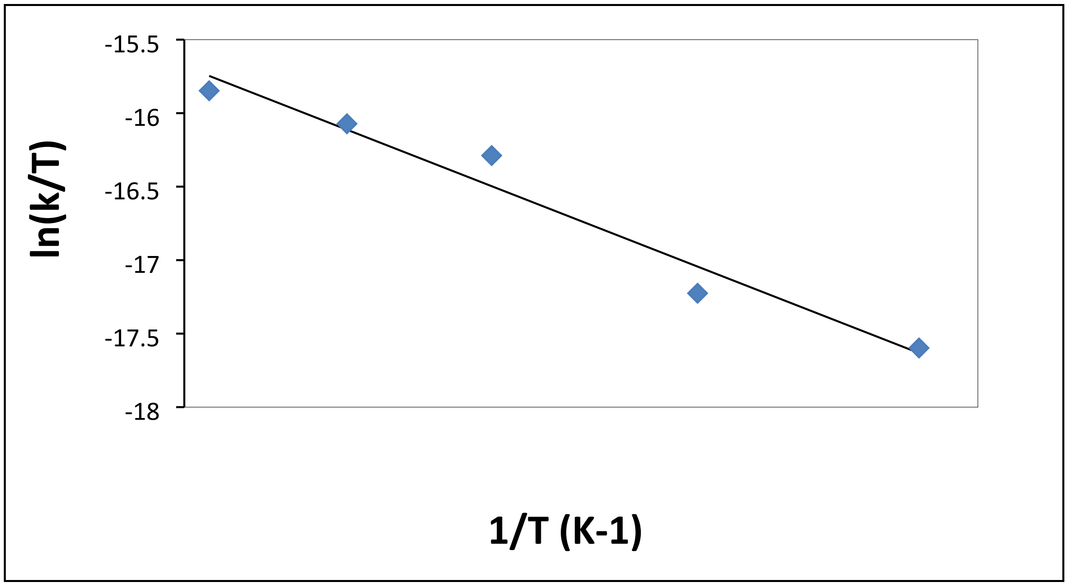

Figure S 13. Eyring plot of aniline insertion into diisopropylcarbodiimide by catalyst **2**.

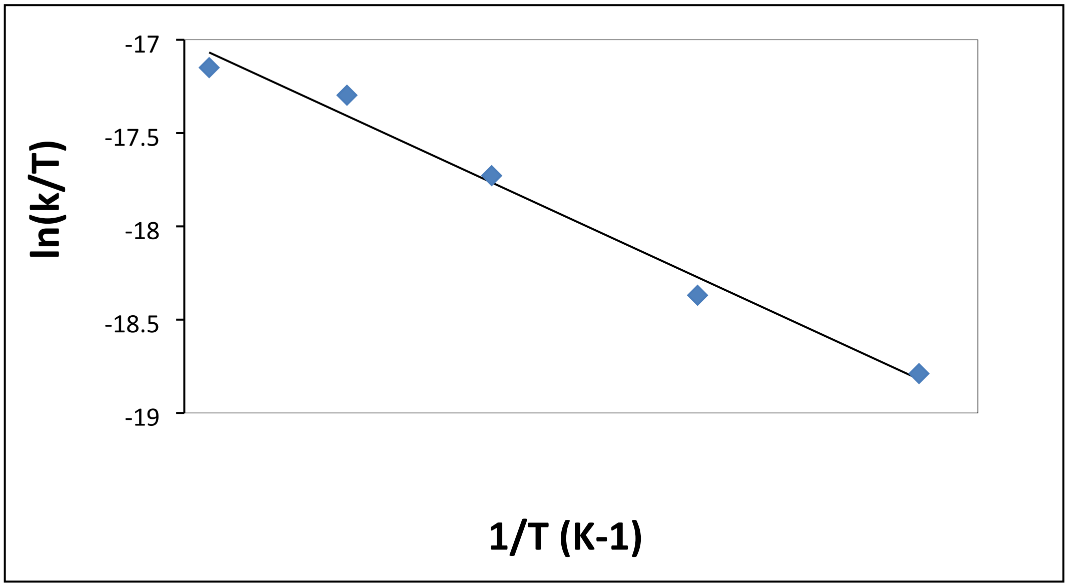

Figure S 14. Eyring plot of aniline insertion into diisopropylcarbodiimide by catalyst **3**.

Kinetic Rate Law

The mechanism provided in **Fig. 5** and rate equation shown in **Eq. 1** is described in more detail below.

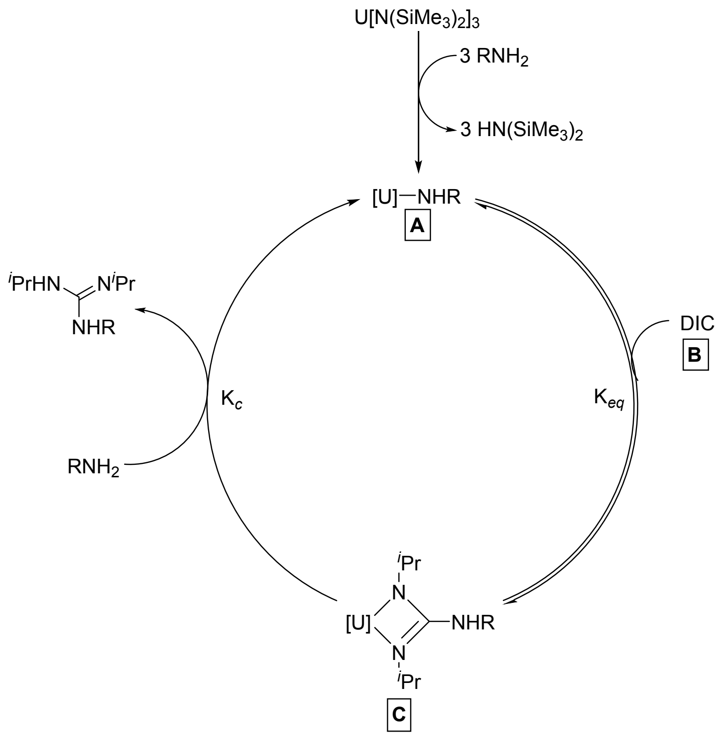

Figure S 15. Catalytic mechanism with intermediates and steps inscribed.

The kinetic rate law for the mechanism depicted is $\frac{\partial P}{\partial T} = K_C[RNH_2][C]$ for the slow protonolysis by amine. In the formation of **C**, the equilibrium equation is A + B $\rightleftharpoons$ C, giving the equilibrium expression $K_{eq} = \frac{[C]}{[A][B]}$. By substituting the equilibrium equation into the kinetic rate law, we receive the following rate law for the full catalytic cycle: $\frac{\partial P}{\partial T} = k_c K_{eq}[A][B][RNH_2]$. This equation reveals the first order kinetics in precatalyst, carbodiimide, and amine.

Crude ^1H NMR spectra of reaction products

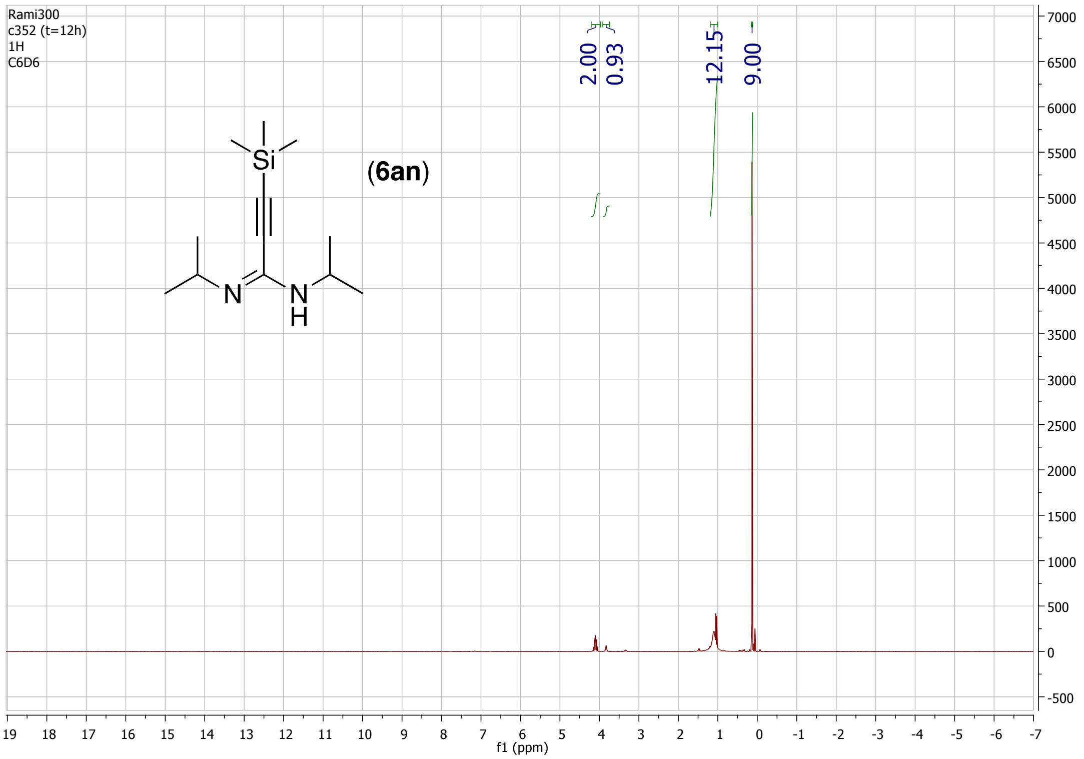

Figure S 16. Crude ^1H-NMR spectrum of **6an**.

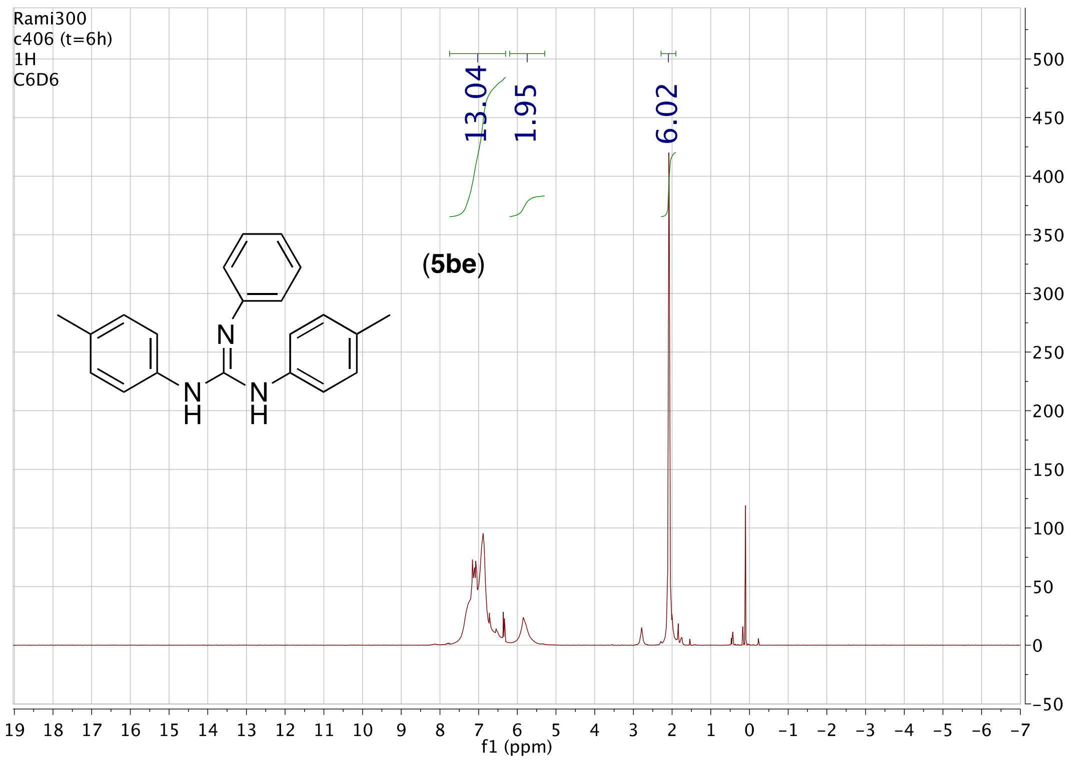

Figure S 17. Crude ^{1}H-NMR spectrum of **5be**.

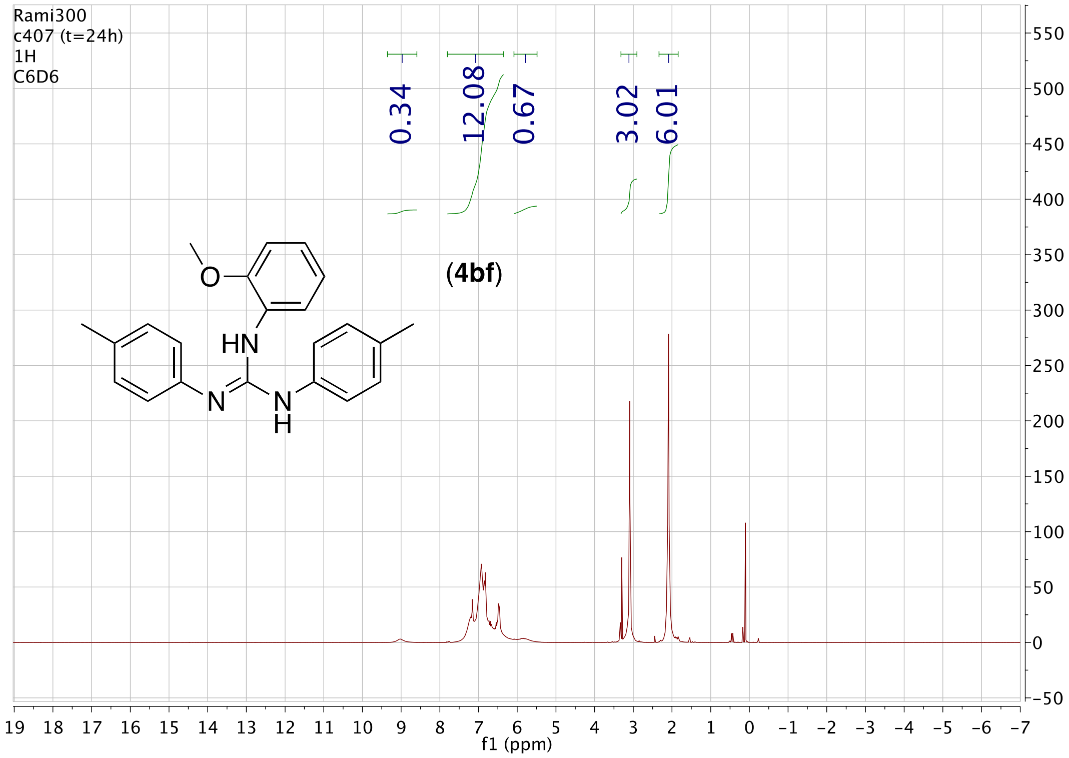

Figure S 18. Crude ^{1}H-NMR spectrum of **4bf**.

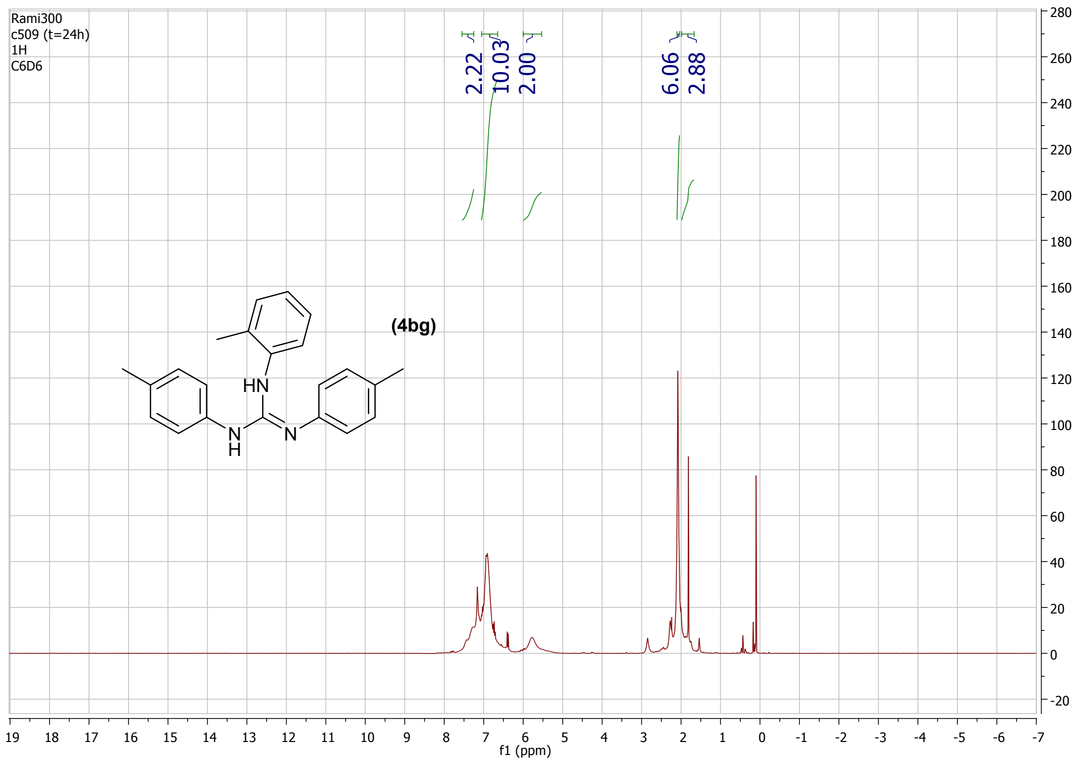

Figure S 19. Crude ^{1}H-NMR spectrum of **4bg**.

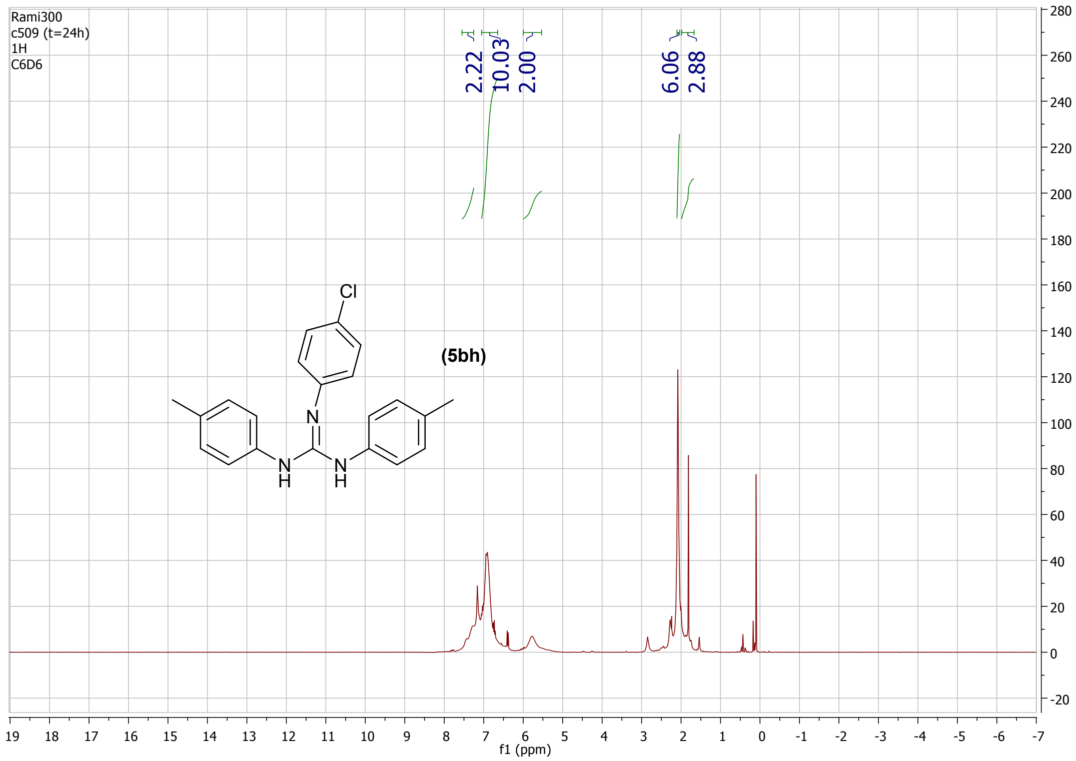

Figure S 20. Crude ^{1}H-NMR spectrum of **5bh**.

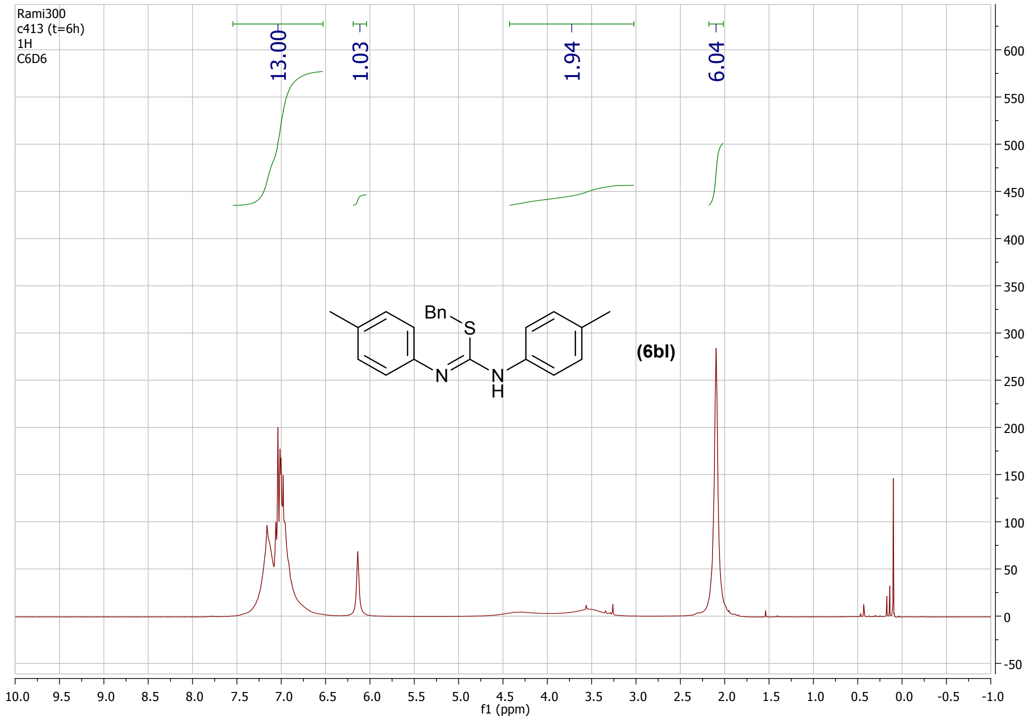

Figure S 21. Crude ^{1}H-NMR spectrum of **6bl**.

Crude ^{13}C NMR spectra of reaction products

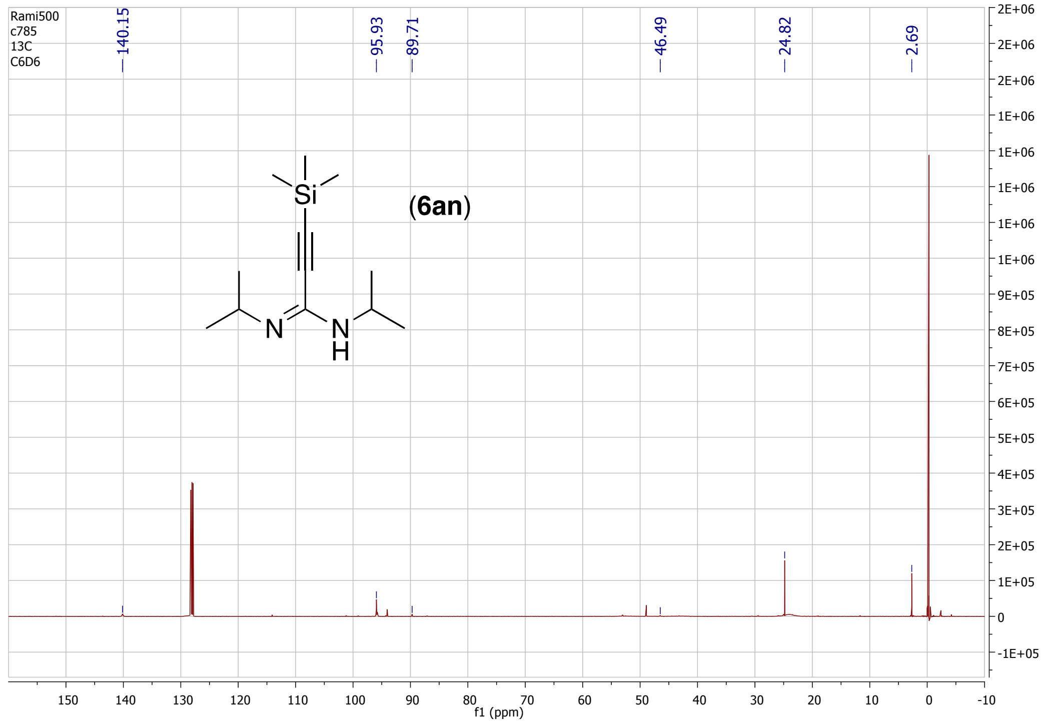

Figure S 22. Crude ^{13}C-NMR spectrum of **6an**.

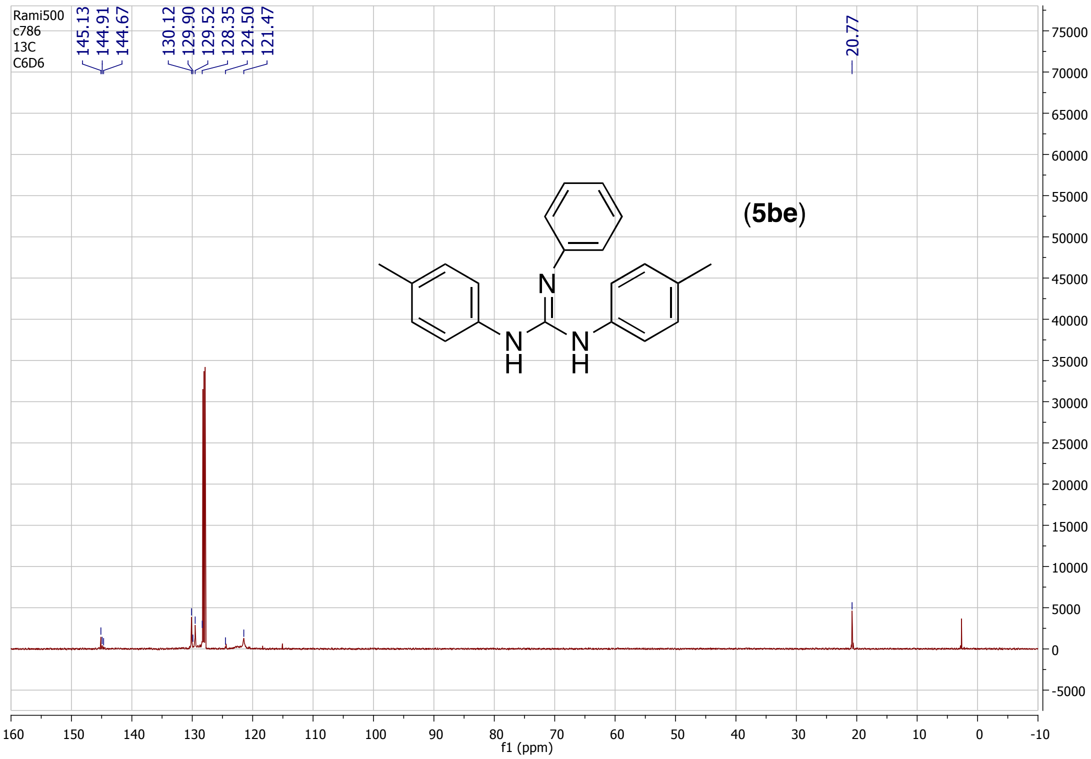

Figure S 23. Crude ^{13}C-NMR spectrum of **5be**.

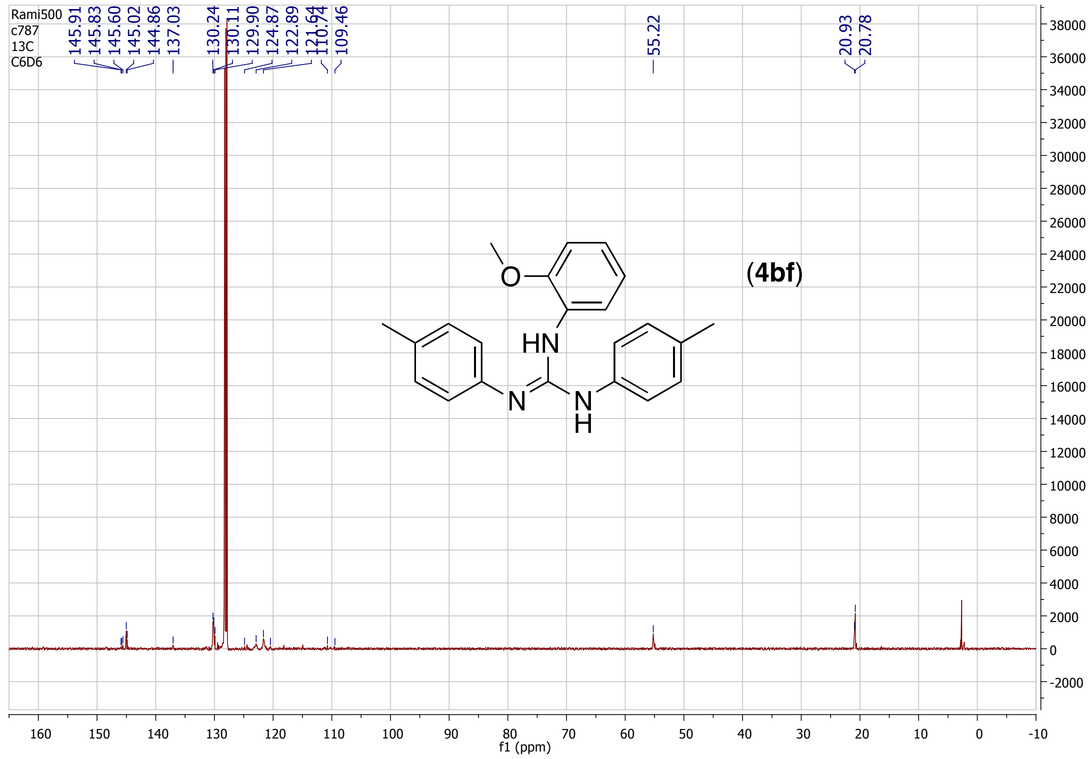

Figure S 24. Crude ^{13}C-NMR spectrum of **4bf**.

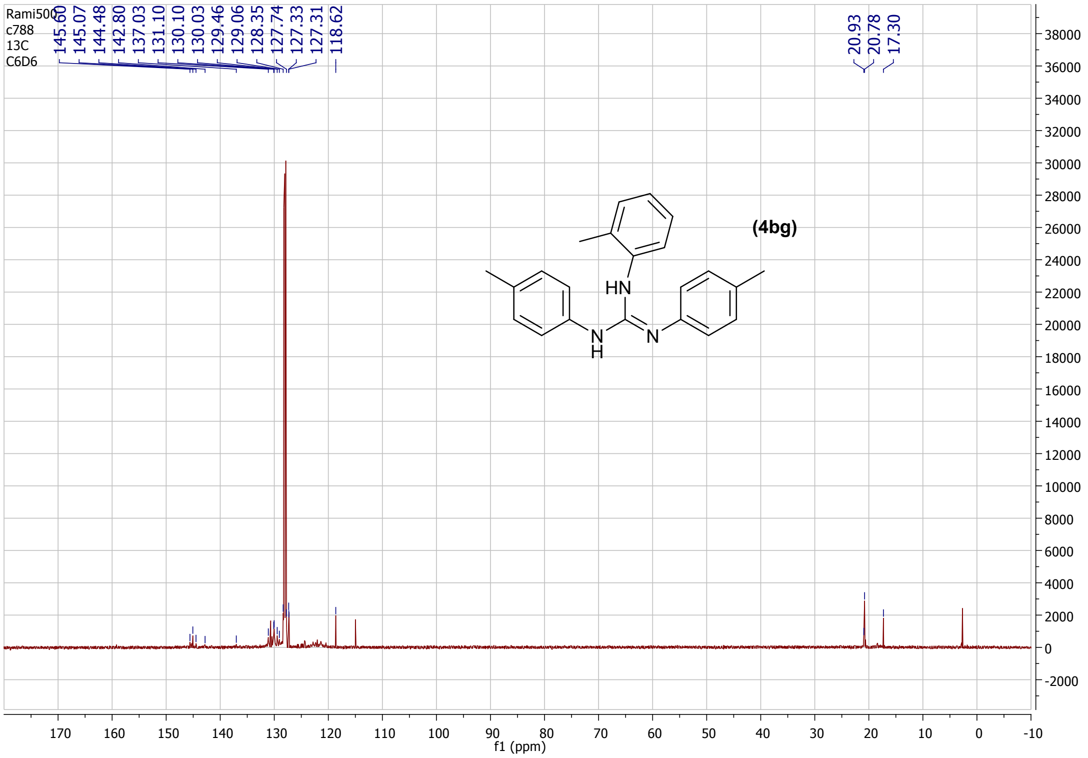

Figure S 25. Crude ^{13}C-NMR spectrum of **4bg**.

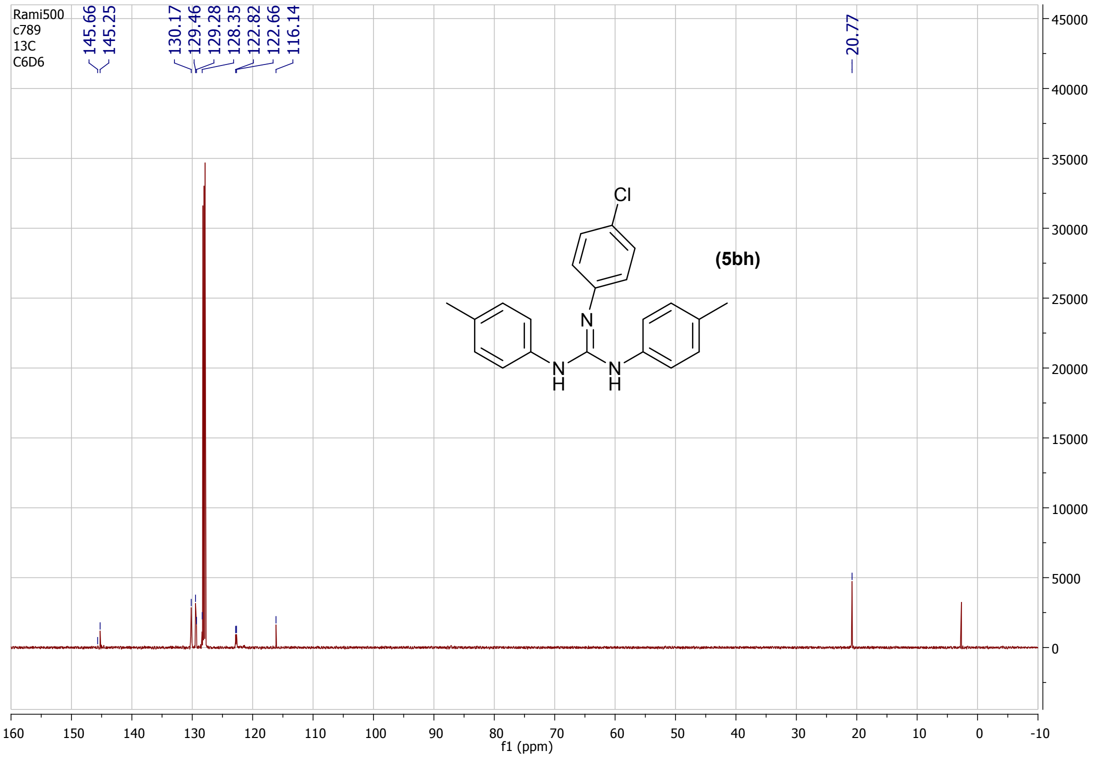

Figure S 26. Crude ^{13}C-NMR spectrum of **5bh**.

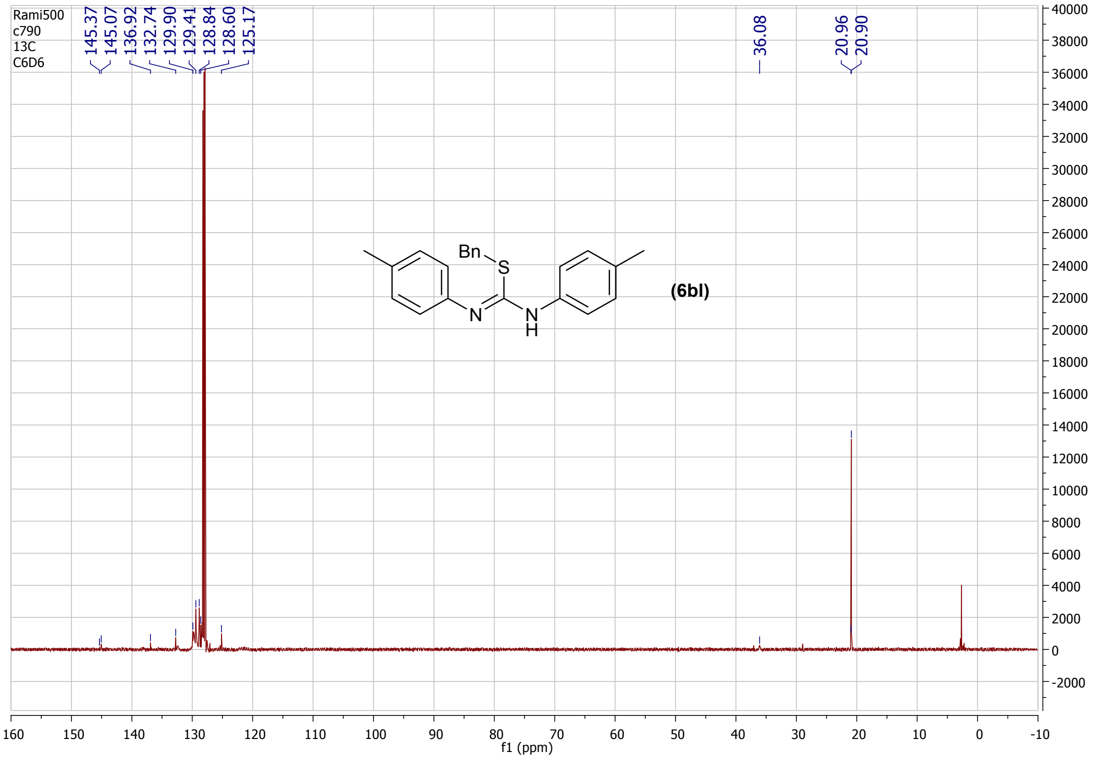

Figure S 27. Crude ^{13}C-NMR spectrum of **6bl**.

REFERENCES

1. R. A. Andersen, *Inorg. Chem.*, 1979, **18**, 1507-1509.
2. M. J. Monreal, R. K. Thomson, T. Cantat, N. E. Travia, B. L. Scott and J. L. Kiplinger, *Organomet.*, 2011, **30**, 2031-2038.
3. T. Cantat, B. L. Scott and J. L. Kiplinger, *Chem. Commun.*, 2010, **46**, 919-921.
4. I. S. R. Karmel, M. Tamm and M. S. Eisen, *Angew. Chem. Int. Ed.*, 2015, DOI: 10.1002/anie.201502041.
5. G. F. Schmidt and G. Süss-Fink, *J. Organomet. Chem.*, 1988, **356**, 207-211.
6. X. Gu, X. Zhu, Y. Wei, S. Wang, S. Zhou, G. Zhang and X. Mu, *Organomet.*, 2014, **33**, 2372-2379.
7. M. R. Crimmin, A. G. M. Barrett, M. S. Hill, P. B. Hitchcock and P. A. Procopiou, *Organomet.*, 2008, **27**, 497-499.
8. A. C. Behrle and J. A. R. Schmidt, *Organometallics*, 2013, **32**, 1141-1149.
9. X. Zhang and S. Lu, *Synlett*, 2005, DOI: 10.1055/s-2005-869839, 1535-1538.
10. S. W. Carr, R. Colton and D. Dakternieks, *Inorg. Chem.*, 1984, **23**, 720-726.
11. V. J. Sattigeri, A. Soni, S. Singhal, S. Khan, M. Pandya, P. Bhateja, T. Mathur, A. Rattan, J. M. Khanna and A. Mehta, *ARKIVOC*, 2005, 46-59.
